# Preclinical modeling of Loeys-Dietz syndrome: insights into mechanisms and therapy

**DOI:** 10.1186/s13023-026-04431-1

**Published:** 2026-06-30

**Authors:** Amira Bousbaa, Ivanna Fedoryshchenko, Ilse Luyckx, Bart Loeys, Aline Verstraeten

**Affiliations:** 1 Center of Medical Genetics, University of Antwerp and Antwerp University Hospital, Antwerp, Belgium; 2 Department of Clinical Genetics, Radboud University Medical Center, Nijmegen, The Netherlands

**Keywords:** Loeys-Dietz syndrome, Thoracic aortic aneurysm and dissection, TGF-β signaling, Mouse modeling, IPSC-derived cell systems

## Abstract

**Background:**

Loeys-Dietz syndrome (LDS) is a rare multisystemic connective tissue disorder characterized by aggressive aortopathy, skeletal, craniofacial, cutaneous and gastrointestinal manifestations. It is caused by pathogenic variants in genes encoding components of the transforming growth factor-beta (TGF-β) signaling pathway, including *TGFBR1, TGFBR2, SMAD2, SMAD3, TGFB2* and *TGFB3*.

**Main body:**

Preclinical modeling has been instrumental in elucidating LDS pathogenesis and informing therapeutic strategies. In this review, a comprehensive overview of available in vivo and in vitro models used to study LDS is presented. We critically evaluate the extent to which each model recapitulates the diverse LDS phenotypes and discuss their respective advantages and limitations. Attention is given to experimental design and variables that influence phenotype expression, including mouse genetic background, sex differences and variant-specific effects. We further synthetize insights gained from these models into the molecular and cellular mechanisms driving LDS, such as the TGF-β paradox, extracellular matrix dysregulation and immune activation. Lastly, we discuss current and emerging therapeutic strategies.

**Conclusion:**

The models described here highlight the critical role of preclinical modeling in advancing our understanding of LDS pathophysiology and therapy, outlining key considerations for future model development and translational application.

**Graphical Abstract:**

**Figure:**
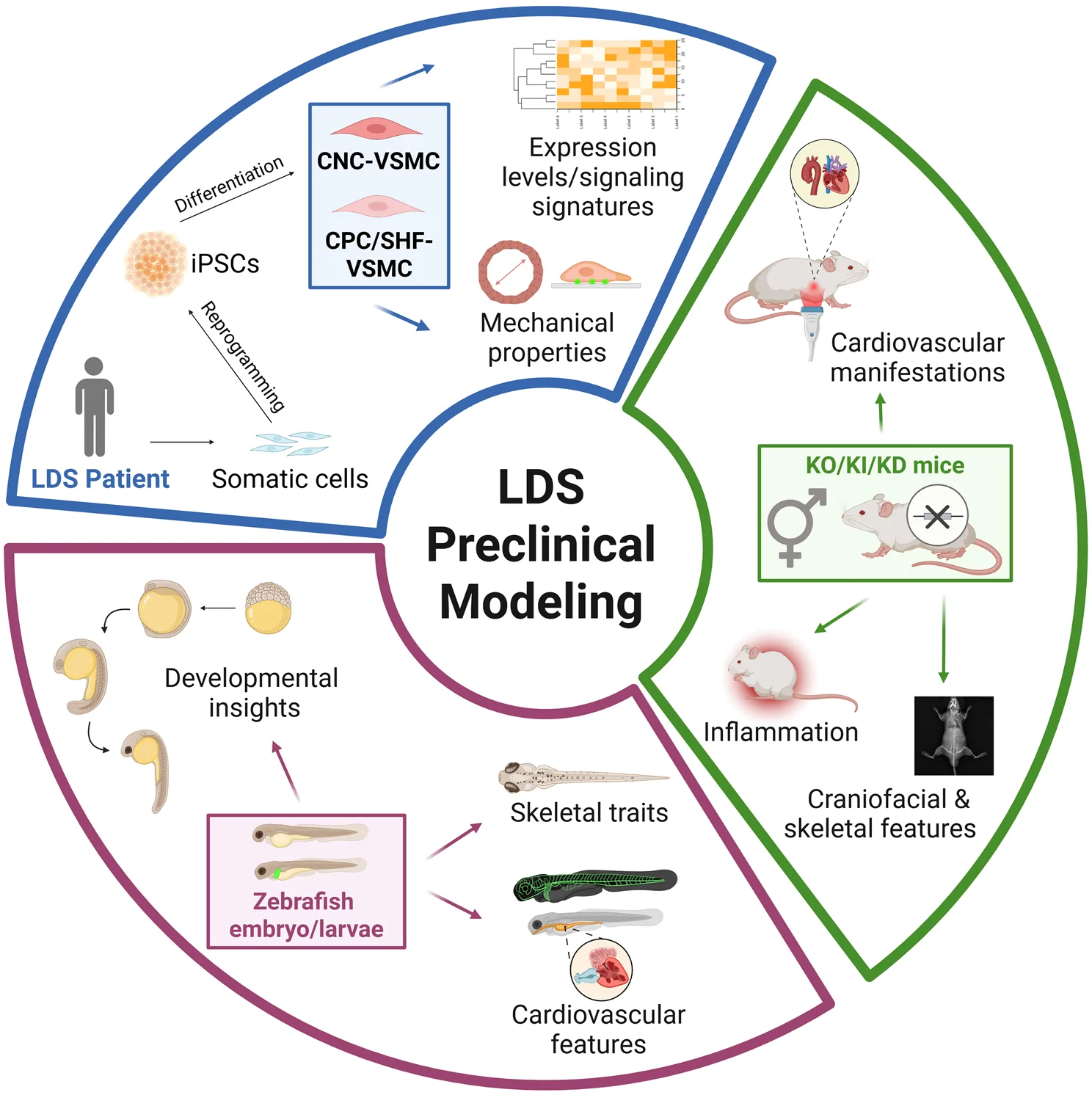

**Supplementary Information:**

The online version contains supplementary material available at https://doi.org/10.1186/s13023-026-04431-1.

## Background

**Fig. 1:**
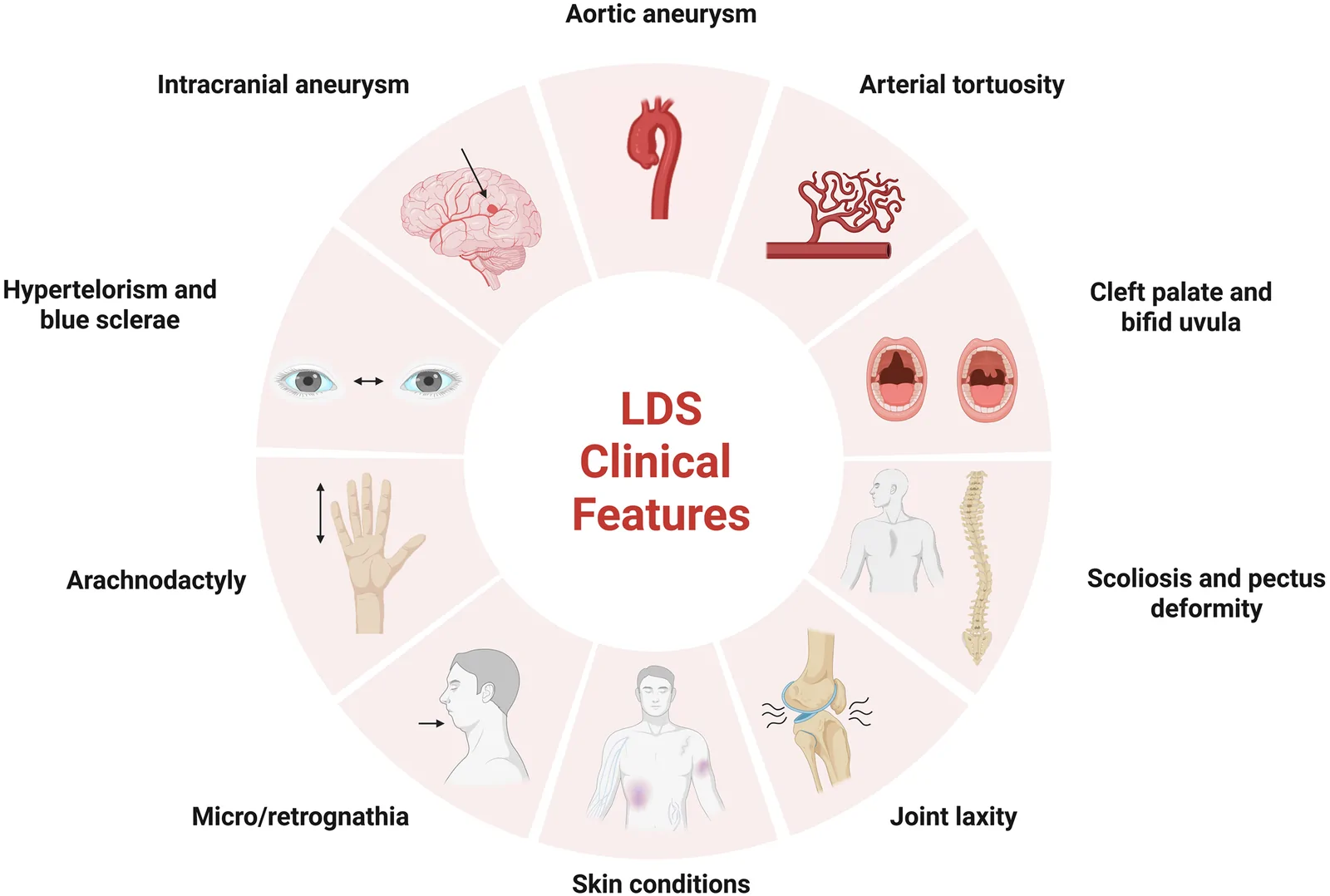
Clinical features of Loeys-Dietz syndrome. Key manifestations span multiple systems: cardiovascular (aortic aneurysm, arterial tortuosity, intracranial aneurysms), craniofacial (hypertelorism, cleft palate, bifid uvula, micro/retrognathia, blue sclerae), musculoskeletal (scoliosis, pectus deformities, arachnodactyly, joint laxity), and cutaneous (easy bruising, translucent skin, visible veins, abnormal scarring, striae).Created in BioRender https://BioRender.com/u87o434

Loeys-Dietz syndrome (LDS) is a monogenic connective tissue disorder first described in 2005, with an estimated prevalence of 1:50,000 [1]. It is characterized by craniofacial, musculoskeletal, cutaneous, gastro-intestinal and cardiovascular abnormalities (Fig. 1) [2]. Common craniofacial symptoms include hypertelorism, bifid uvula, a cleft or high-arched palate, enamel defects, retrognathia and craniosynostosis [1–4]. With respect to the musculoskeletal system, pectus deformity, scoliosis and joint laxity are frequently observed [1, 5, 6]. A velvety, translucent skin with visible veins, as well as easy bruising and dystrophic scars are recurrent skin findings [1, 6], while eosinophilic gastro-intestinal disease and inflammatory bowel disease are among the gastro-intestinal complications [3]. Most critically, LDS patients frequently present with vascular manifestations associated with high morbidity and mortality rates, including aortic/arterial tortuosity, aneurysms and dissections throughout the arterial tree, necessitating regular head-to-pelvis imaging [2]. Cardiac abnormalities such as bicuspid aortic valve, patent ductus arteriosus and atrial septal defects are more commonly observed than in the general population.

**Fig. 2:**
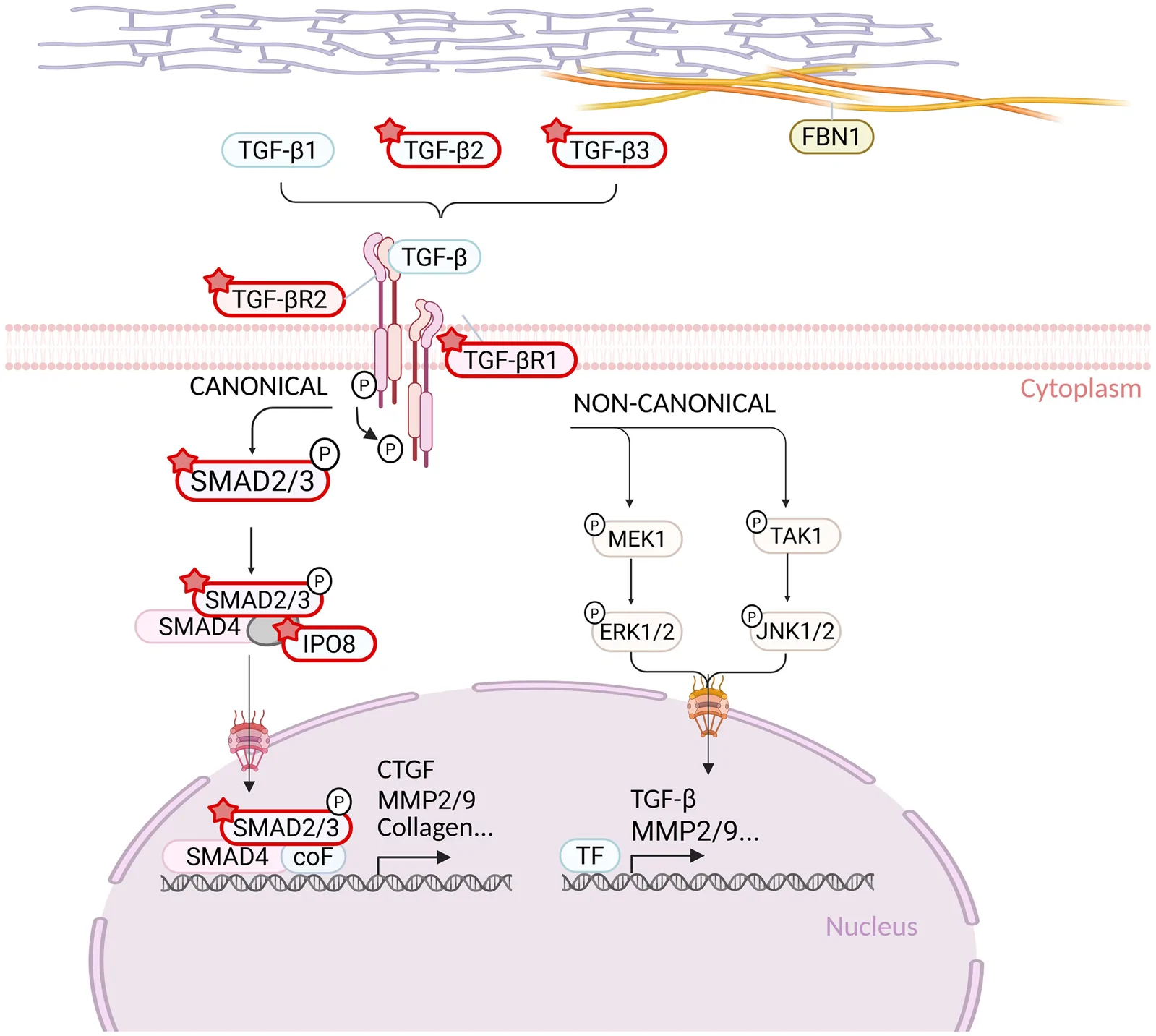
TGF-β signaling pathway. The genes *TGFB1*, *TGFB2* and *TGFB3* encode three isoforms of the TGF-β ligands, namely TGF-β1, TGF-β2 and TGF-β3. TGF-β is released from cells in the form of a large latent complex, including the mature TGF-β cytokine, a dimer of its processed amino-terminal propeptide (LAP) and one of three latent TGF-β binding protein isoforms (LTBP1, 3, or 4). This complex binds to ECM components. After its release from this complex, TGF-β attaches to the type II TGF-β receptor subunit (TGF-βR2), inducing a change in its conformation and the phosphorylation of the type I TGF-β receptor subunit (TGF-βR1). In the canonical pathway, TGF-βR1 phosphorylates SMAD2 and SMAD3 proteins, which then associate with SMAD4 and translocate to the nucleus to regulate TGFβ-mediated gene transcription. Additionally, ligand-activated TGF-β receptors can initiate non-canonical signaling cascades, including RhoA and mitogen-activated protein kinases such as ERK, JNK and p38 [7, 8]. Pathogenic variants in *TGFBR1, TGFBR2, SMAD3, TGFB2, TGFB3* and *SMAD2* cause Loeys-Dietz syndrome types 1, 2, 3, 4, 5 and 6, respectively. Created in BioRender https://BioRender.com/u87o434

LDS is caused by pathogenic variants in genes encoding key components of the transforming growth factor (TGF)-β pathway, i.e., ligands (*TGFB2/3*), receptors (*TGFBR1/2*) and downstream effectors (*SMAD2/3*) (Fig. 2, Table 1) [1, 9–12]. TGF-β signaling plays an important role in cellular processes such as inflammation, cell differentiation, tissue homeostasis, vascular remodeling and embryogenesis [15]. Pathogenic loss-of-function (LoF) variants in LDS genes result in a paradoxical upregulation of the TGF-β pathway at the tissue level, impairing extracellular matrix (ECM) homeostasis and thus predisposing to connective tissue abnormalities [16]. Based on the affected gene, LDS is categorized into six subtypes (Table 1) [3]. Pathogenic variants in *TGFBR1* and *TGFBR2* (LDS type 1 & 2) were first identified and are, to date, still considered to result in the most severe and penetrant LDS phenotypes. LDS3-6 more commonly present with reduced penetrance and variable expressivity, with LDS5 (*TGFB3*) residing at the mildest end of the spectrum [17]. Notably, an autosomal recessive disorder caused by biallelic variants in *IPO8*, encoding importin‑8, a mediator of nuclear transport of activated SMADs, first described in 2021, closely resembles the established autosomal dominant LDS subtypes but has not yet been officially classified as part of the LDS spectrum (Table 1) [13]. Given the striking clinical and pathophysiological overlap, IPO8-related syndrome has also been included in this review. Intrafamilial variability is common in LDS. Genetic modifiers are thought to contribute to this variability, although their involvement remains largely unexplored [18, 19].

**Table 1:** Loeys-Dietz syndrome: phenotype description by subtype

Subtype	Gene	OMIM	Penetrance	Distinct features	Inheritance	Ref
LDS1	*TGFBR1*	609192	High	Aneurysms in the aorta and arterial branches, craniofacial features (hypertelorism, bifid uvula/cleft palate).	Autosomal dominant	[1]
LDS2	*TGFBR2*	610168	High	Similar to LDS 1, but potentially more severe arterial tortuosity and higher risk of dissection.	Autosomal dominant	[1]
LDS3	*SMAD3*	613795	High to Moderate	Early-onset osteoarthritis, mitral valve prolapse, aortic aneurysm/dissection and arterial tortuosity.	Autosomal dominant	[9]
LDS4	*TGFB2*	614816	Moderate	Thoracic aortic aneurysms, skeletal findings, mild craniofacial involvement.	Autosomal dominant	[10]
LDS5	*TGFB3*	615582	Low	Similar to LDS 4; lower risk of aneurysm dissection.	Autosomal dominant	[11]
LDS6	*SMAD2*	619656	Moderate to Low	Aortic and arterial aneurysms/dissections, arterial tortuosity, congenital heart disease.	Autosomal dominant	[12]
*IPO8* syndrome^a^	*IPO8*	619472	High	Severe early-onset aortic aneurysm, motor development delay, craniofacial involvement.	Autosomal recessive	[13]

*IPO8 aortopathy is not yet officially classified as an LDS subtype in OMIM catalog, but included in the most recent Genereviews* [14]

LDS management includes symptomatic drug treatment, prophylactic measures, genetic counseling and lifestyle changes [3]. With respect to the life-threatening vascular manifestations, drug therapy with β-blockers (e.g., propranolol) and/or angiotensin-II-receptor antagonists (e.g., losartan) aims to decrease hemodynamic stress and/or to address the remodeling processes impacting the aortic wall [20]. β-blockers lower the heart rate, while angiotensin-II-receptor antagonists both reduce blood pressure and regulate TGF-β signaling. Both therapeutic avenues slow down aneurysm progression but cannot fully prevent dissections and/or ruptures [21]. Consequently, yearly echocardiography and/or MRA/CT angio-imaging is recommended to detect early signs of worrisome aneurysm progression [3]. Prophylactic surgery is generally considered when the aortic root diameter exceeds 45 mm, but other factors such as the nature of the pathogenic variant, family history of dissection and aortic growth rate per year can influence the surgical threshold [20, 22]. Management strategies have also been established for complications affecting other organ systems and are aligned with standard care applied in non‑LDS patients with the same clinical features [20, 22].

Comprehensive preclinical research into disease mechanisms, coupled with rigorous drug testing using robust and clinically relevant model systems, is essential for the development of more effective, pathophysiology-driven and personalized therapeutic interventions. Despite some limitations, existing LDS model systems have been shown to recapitulate the diverse manifestations affecting the various involved organ systems, rendering them suitable for impactful preclinical research [23–29]. Here, we review and critically reflect on the current literature and future perspectives in preclinical LDS research.

## Preclinical LDS models

### In vivo models

Animal models have long been, and still are, central to studying LDS pathology. The relevance of these models lies in their anatomic and phylogenetic similarity to humans, as well as their ability to replicate cell-cell/organ-organ communication and dynamic disease processes. Mouse models are the most extensively used and characterized systems in LDS research, as the human organs that are highly enriched in connective tissue closely resemble the murine counterparts [30, 31]. They have been used to study nearly all phenotypic features, with a focus on the cardiovascular phenotype.

Zebrafish have emerged as a popular alternative, offering several advantages, including high fecundity, transparent embryonic development, short generation time and relatively high degree of genome homology with humans. Approximately, 70% of human genes possess at least one known ortholog, compared to ~80% in mouse [32, 33].

#### Mouse models

Mice harboring LoF variants in LDS genes have been developed on various backgrounds, predominantly 129/Sv and C57BL/6 (Table 2). The cardiovascular system has been most thoroughly characterized, whereas other manifestations, such as craniofacial and musculo-skeletal abnormalities, are inconsistently assessed and only occasionally documented.

**Table 2:** Overview of Loeys-Dietz syndrome in vivo and in vitro preclinical models

Model	Gene(s)	Variant(s)^a^	Background(s)	Gender^b^	Phenotype	Ref
Mouse	*Tgfb2*	Heterozygous knockout	Mixed background (unknwon)	N/A	Dilatation of the aortic annulus and root starting from 4 months of age, reaching aneurysms by 8 months, increased collagen deposition and disorganized elastic fibers. Normal-sized ascending aorta at 8 months of age.	[34]
	*Tgfb2*	Homozygous and heterozygous knockout (exon 6)	129/Ola × BL(S)	M & F	*Tgfb2*^-/-^: embryonic and perinatal lethality (2/3 of mutants). E18.5 fetuses exhibit structural cardiac, craniofacial, skeletal, eye and ear defects. *Tgfb2*^+/-^: no significant embryonic lethality, no apparent defects compared to WT.	[35]
	*Tgfb2*	Homozygous knockout	129/Ola × BL(S) Generated as described in [35]	N/A	Mutant embryos present with multiple cardiovascular abnormalities between E11.5-18.5 (double-outlet right ventricle, thickening of semilunar valves, ventricular septal defects and atrioventricular canal malformations, hypoplasia or interruption of aortic segments, hypoplastic pulmonary arteries).	[36]
	*Tgfb2*	Homozygous knockout-first (intron 1) and systemic conditional KO (exon 2)	C57BL/6	N/A	*Tgfb2*^*KOf/-*^ and *Tgfb2*^*cKO/-*^: highly penetrant perinatal lethality, multiple congenital heart defects (double-outlet right ventricle, thick aortic and pulmonary valves, double-inlet left ventricle, overlapping of tricuspid valve orifices, abnormal morphology of tricuspid and mitral valves). Overall phenotype is similar to that of full-KO *Tgfb2*^-/-^ mice.	[37]
	*Tgfb3*	Homozygous knockout (exon 6)	C57BL/6 and BL(S)	N/A	Complete penetrant perinatal lethality within 11-20 h of birth, delayed pulmonary development, palatal clefting. No obvious structural heart or skeletal abnormalities.	[38]
	*Tgfb3*	Homozygous and heterozygous knockout (exon 6)	C57BL/6, 129/Ola and CF-1	N/A	*Tgfb3*^-/-^: Highly penetrant perinatal lethality within ~24 h of birth, no obvious malformations in the heart, brain, skeletal, or cartilage. Mutants exhibit palatal clefting (anterior or posterior), with greater severity (complete clefting) and incidence on C57BL/6 background. *Tgfb3*^+/-^: no apparent phenotype.	[39]
	*Tgfb3*	Homozygous and heterozygous knockout (exon 6)	Mice obtained from [39] and maintained on C57BL/6	N/A	*Tgfb3*^-/-^: mutant fetuses display multiple cardiovascular anomalies (cardiac OFT and atrioventricular canal defects). *Tgfb3*^+/-^: mild cardiovascular defects at E14.5.	[40]
	*Tgfb1/2/3*	Heterozygous knockout	C57BL/6	M & F	*Tgfb2*: altered aortic arch curvature and right innominate branch artery, significant thickening of the aortic wall without any dilatation. Only *Tgfb2* mutants differed significantly upon biomechanical testing (biaxial stress and stiffness). *Tgfb1/3*: few aortic structural differences (shorter aorta with reduced arch curvature) compared to WT animals.	[41]
	*Tgfbr1*	Heterozygous missense variant c.953T > G; p.M318R	129S6/SvEv Same experimental animals/research group as [25]	M & F	Dilated aortic root from 16 weeks of age, impaired aortic architecture, defective activation of the canonical TGF-β pathway only in SHF-derived VSMCs compared to CNC-VSMCs.	[26]
	*Tgfbr1*	Homozygous and heterozygous missense variant c.563 G > T; p.G188V	C57BL/6	M	*Tgfbr1*^*G188V/G188V*^: embryonically lethal after E14.5 (only one mutant survived until birth). *Tgfbr1*^*G188V/+*^: distorted and torn aortic elastic fibers at 24 weeks, tendency towards premature death compared to WT mice. No obvious periodontal tissue abnormalities or alveolar bone resorption at 6 and 24 weeks. Higher susceptibility to bacterial-induced periodontitis compared to WT.	[42]
	*Tgfbr1*	Homozygous and heterozygous nonsense variant c.1134C > A; p.Y378* (exon 7)	C57BL/6	M & F	*Tgfbr1*^Y378*/Y378*^: early embryonic lethality due to defective vascularization. *Tgfbr1*^Y378*/+^: no significant differences in development, lifespan, elastic fiber structure and TGF-β signaling.	[43]
	*Tgfbr1*	Late postnatal (7-15-week-old) homozygous VSMC-specific deletion (*Myh11*-Cre; exon 3)	C57BL/6	M & F	Dimorphic incidence of aortic aneurysms, female and male hormones had protective and limited effect on the phenotype, respectively. Female *Tgfbr1*^*flox/flox*^: little to no signs of gross aortopathy. Male *Tgfbr1*^*flox/flox*^: severe aortopathy (aortic layer degeneration, aortic dilatation and rupture).	[44]
	*Tgfbr1/2*	Heterozygous missense variants *Tgfbr1* (c.953T > G; p.M318R), *Tgfbr2* (c.1069 G > T; p.G357W) and haploinsufficient alleles (exon 7 and 4, resp.)	129S6/SvEv	N/A	*Tgfbr1*^*M318/+*^ *and Tgfbr2*^*G357W/+*^: accelerated aortic root growth, deteriorated aortic architecture (wall thickening, increased collagen deposition and fragmentation of elastic fibers), coronary artery tortuosity, thoracic kyphosis and severe craniosynostosis with partial coronal synostosis. *Tgfbr1*^+/-^ and *Tgfbr2*^+/-^: comparable aortic root size and growth to WT animals, without any other features.	[25]
	*Tgfbr1/2*	Heterozygous missense variants *Tgfbr1* (c.953T > G; p.M318R), *Tgfbr2* (c.1069 G > T; p.G357W)	Mice model from [25]	M & F	*Tgfbr1*^*M318/+*^: normal enamel rod decussation pattern, smooth enamel surface. *Tgfbr2*^*G357W/+*^: fully penetrant structural enamel defects (altered attrition pattern, severe alteration in the enamel rods arrangement pattern, decreased enamel hardness). No significant changes in enamel mineralization compared to controls. Ameloblasts showed altered cytoskeleton architecture and altered coordinated movement, with abnormal tight-junction (ZO-1) distribution at the ATW. Unaffected SMAD2/3 activation.	[45, 46]
	*Tgfbr1/2*	Heterozygous missense variants *Tgfbr1* (c.953T > G; p.M318R), *Tgfbr2* (c.1069 G > T; p.G357W)	129/SvEv	N/A	Allergic inflammatory phenotype (eosinophilic esophagitis features).	[47]
	*Tgfbr2*	Heterozygous missense variant c.1069 G > T; p.G357W	129S6/SvEv	F	Altered bone morphology (reduced cross-sectional tissue, bone area and cortical thickness) as well as impaired bone strength.	[48]
	*Tgfbr2*	Heterozygous missense variant c.1069 G > T; p.G357W	129S6 Mice obtained from [25]	M & F	Cranial doming, anterior shortening of the cranium as early as 2 weeks, nasal bones asymmetry, palatal and cranial suture defects, shortening of the mandibles … etc. Variable mandibular size reduction among mutants, cranial doming and severe nasal asymmetry were more frequent in females.	[49]
	*Tgfbr2*	Early postnatal (6-week old) homozygous VSMC-specific deletion (*Acta2*- and *Myh11*-Cre; exon 4)	C57BL/6	M & F	Major disruption of aortic wall structure (intramural hemorrhage, wall thickening, ulceration … etc.), penetrant aortic dilatation at 14 weeks post-deletion (mainly ascending segment, descending or abdominal regions in some cases), increased VSMC contractile gene expression (mRNA).	[50]
	*Tgfbr2*	Early postnatal (4-week old) homozygous and heterozygous VSMC-specific deletion (*Myh11*-Cre; exon 2)	C57BL/6	N/A	*Tgfbr2*^*flox/flox*^: penetrant dilatation of the ascending aorta by 6 weeks of age, aortic dissections (~50% of mutants by 8 weeks of age), hemorrhage and thickening of the aortic wall, fiber fragmentation, adventitial fibrosis, medial atrophy, immune cell infiltration, altered TGF-β signaling and sensitivity (increased non-canonical and reduced canonical signaling), absence of aortic ruptures. *Tgfbr2*^*flox/+*^: absence of aortic disease 2–4 weeks post-deletion, similar but attenuated alterations in TGF-β signaling.	[51]
	*Tgfbr2*	Early postnatal (6-week old) homozygous VSMC-specific deletion (*Myh11*-Cre; exon 4)	C57BL/6J	M	VSMC hypercontractility, severe endothelial dysfunction (impaired relaxation responses, reduced levels of aortic NO and eNOS). Absence of immune cell infiltration, intramural hemorrhage, elastolysis and aortic dilatations at 1 week post-deletion.	[52]
	*Tgfbr2*	Late postnatal (11-month-old) homozygous VSMC-specific deletion (*Acta2*-Cre; exon 4)	C57BL/6J	M & F	Mild aortic histopathology (increased medial area and thickness) and altered vasomotor function. Absence of aortic dilatation, dissection or rupture.	[53]
	*Tgfbr1/2*	Late postnatal (9-13-week-old) homozygous VSMC-specific deletion (*Myh11*-Cre; exon 3 and 2, resp.)	C57BL/6	M	*Tgfbr1*^*iko*^: rapid ascending aortic aneurysmal disease with complete penetrance (aortic enlargement, elastic fiber fragmentation, medial layer tear). *Tgfbr2*^*iko*^: mild aortopathy with low penetrance compared to mutants *Tgfbr1*^*iko*^.	[28]
	*Tgfbr2*	Conditional homozygous cranial NC-specific deletion (*Wnt1*-Cre; exon 2)	C57BL/6J	N/A	Complete secondary palate clefting at E14.5, calvaria agenesis, skull defects (absence of frontal and poorly developed parietal bone, small mandible and maxilla) at full penetrance.	[54]
	*Tgfbr2*	Conditional homozygous NC-specific deletion (*Wnt1*-Cre; exon 2)	N/A	N/A	Normal NC number, migration pattern and specification to VSMC fate in both cranial and cardiac regions at E10.5-E11.5, fully penetrant cardiovascular abnormalities from E12.5 (persistent truncus arteriosus, absent aortic-pulmonary septum and interrupted aortic arch).	[55]
	*Tgfbr2*	Homozygous SHF-specific deletion (*Mef2c*-Cre; exon 4)	C57BL/6J	M	Embryonic lethality at E12.5, dilatation of the OFT and retroperitoneal hemorrhages.	[56]
	*Smad2*	Homozygous and heterozygous knockout	C57BL/6 × BL(S)	N/A	*Smad2*^-/-^: embryonic lethality before E8.5, abnormal egg cylinder formation, failed gastrulation and mesodermal induction. *Smad2*^+/-^: viable, fertile, comparable in size and growth to WT.	[57]
	*Smad2*	Homozygous NC-specific knockout (*Wnt1*-Cre; exon 2)	C57BL/6	M & F	Viable, defective differentiation of NC-VSMCs in aortic arch arteries but not in dorsal aorta during embryonic development, abnormal structure of adult carotid arteries (lack of one VSMC layer in the media of arteries, thin elastic lamina).	[58]
	*Smad3*	Homozygous and heterozygous knockout (exon 2)	129Sv and 129Sv × C57BL/6	M & F	*Smad3*^+/-^: viable and fertile. No obvious phenotype up to 9 months. *Smad3*^-/-^: viable, small-sized compared to WT (especially males). Mutants show colonic obstruction and spontaneous colorectal tumor formation between 18 and 24 weeks of age. Reduced penetrance of the tumor phenotype on the mixed background.	[59]
	*Smad3*	Homozygous knockout (exon1-intron1)	129Sv × C57BL/6	N/A	No embryonic lethality, normal survival to adulthood. Mutants are small-sized and develop forelimb malformations (and/or kyphosis with rib cage malformation). Increased proliferation of *Smad3-*null-derived embryonic fibroblasts compared to WT at baseline, defective TGF-β signaling in *Smad3-*null-derived immune cells.	[60]
	*Smad3*	Homozygous knockout (exon 8)	129SvEv × C57BL/6 and 129SvEv × BL(S)	N/A	No embryonic lethality, 70% of mutants have growth retardation and develop a wasting syndrome from 3 weeks of age. Mutants present with immune dysregulation and inflammatory lesions affecting the stomach, mucosa, pancreas, colon and small intestine leading to death (1–8 months). Similar phenotypes on both backgrounds.	[61]
	*Smad3*	Homozygous knockout (exon 8)	Mixed background (unknown)	N/A	Small-sized mutants, spine anomalies, inter-vertebral degeneration and calcification (decreased chondrocyte height, decreased collagen and proteoglycans content).	[62]
	*Smad3*	Homozygous knockout (exon 8)	Mixed background (Animals/research group [61])	N/A	Degenerative joint disease (loss of articular cartilage, decreased production of proteoglycans).	[63]
	*Smad3*	Homozygous and heterozygous knockout (exon 8)	C57BL/6 Generated as described in [61]	N/A	*Smad3*^-/-^: early onset (2 months) and rapid progression of aortic root aneurysm (ascending aorta dilation and/or rupture between 4 and 8 months), immune cell infiltration and increased expression of matrix-degrading MMPs. *Smad3*^+/-^: viable, delayed aortic root dilation (~8 months).	[64]
	*Smad3*	Homozygous knockout	C57BL/6	M & F	Early onset (6 weeks) and rapid progression of aneurysms (aortic root and ascending aorta), early and sudden death before 3 months. Adventitial inflammation and medial neovascularization. Increased activity of MMPs in inflammatory regions and VSMC proliferation. No ECM or collagen accumulation. Aneurysmal phenotype is more severe in males, with more premature death (65% in males vs. 22% in females).	[65]
	*Smad3*	Early postnatal (6-week-old) homozygous and heterozygous VSMC-specific knockout (*Myh11*-Cre; exon 2-3)	129S6/SvEv	M	*Smad3*^*flox/+*^: no sign of aortic dilatation (~18 weeks post-deletion). *Smad3*^*flox/flox*^: significant aortic dilatation of the aortic root (18 weeks post-deletion), impaired expression of ECM proteins, loss of contact between VSMC layer and elastic lamella (10 weeks post-deletion).	[24]
	*Ipo8*	Homozygous knockout (exon 1-14)	C57BL/6N	M & F	Dilatation of the aortic root, fragmentation and disorganization of elastic fibers in the aortic wall. Males showed more pronounced aortic phenotype (aortic ruptures), as well as unique and severe dilatated ascending segments.	[13]
Zebrafish	*tgfbr1*	Homozygous frameshift variants alk5a (exon 1) and/or alk5b (exon 4)	N/A	*alka*^-/-^ and *alk5b*^-/-^: no phenotype. *alk5a/b*^-/-^: (double mutants): defective cardiac development, severe dilatation of the OFT, endothelial layer tear, abnormal elastin structure, impaired TGF-β signaling.		[66]
	*tgfbr2b*	Homozygous knockdown (exon 7)	N/A	Developmental delays in the head and tail, craniofacial dysmorphism (small and dislocated eyes, small bent bodies, short caudal fin … etc) and cardiovascular defects (cardiac effusion, linear cardiac chambers).		[67]
	*smad3a*	Homozygous missense variant c.730 G > T; p.V244F	N/A	Increased diameter of the dorsal aorta at 48 hpf.		[68]
	*ipo8*	Homozygous frameshift variant (exon 4)	N/A	Severely impaired dorso-ventral patterning, impaired tail extension, cardiovascular abnormalities (heart edema, arterio-venous malformations in the head, irregular and poorly formed central arteries).		[69]
Heterologous and patient-derived cell systems	*TGFBR1*	Several heterozygous missense variants	F	HEK293T cell line Significant reduction in luciferase reporter gene activity, abolished SMAD2 nuclear localization, downregulated TGF-β signaling upon stimulation.		[70]
	*TGFBR1*	Heterozygous missense variant c.1126A > G; p.(K376E)	F	HEK293T cell line Abolished reporter activity in mutant cells after treatment with recombinant TGFB1. Elevated levels of pSMAD2 in patient’s aortic tissue.		[71]
	*TGFBR2*	Heterozygous missense variants c.1613T > C; p.V538A and c.1255 G > T; p.V419L	F	HEK293T cell line Reduced canonical TGF-β signaling (pSMAD2/3).		[72, 73]
	*TGFBR2*	Heterozygous missense variant c.1069 G > T; p.G357W	N/A	Primary HAoSMC Transduced VSMCs induced increased MMP activity and impaired migration in a wound healing assay, obvious loss of filamentous actin, reduced contractile protein expression.		[74]
	*SMAD3*	Heterozygous missense variant c.1262 G > A; p.(C421Y)	F	HEK293T cell line Impaired TGF-β pathway activation upon TGFB1 stimulation, significantly lower phosphorylation levels of SMAD3. Increased levels of pSMAD3 in patient’s aortic tissue.		[75]
	*SMAD3*	Heterozygous DN variants c.859C > T; p.(R287W) and c.802C > T; p.(R268C) and HI variant c.741-742delAT; p.(F248Pfs*62)	M & F	Patient-derived fibroblasts and VSMC Patient-transdifferentiated myofibroblasts: increased differentiation response in the presence of TGFB1, higher expression of contractile markers of HI variant, compared to DN variants. DN mutants had disorganized and delayed FBN1-ECM. Unaltered pSMAD2/3 levels in both conditions. Patient-derived VSMCs: Similar contractile markers expression in HI mutant VSMCs compared to myofibroblasts. DN mutant VSMC have higher expression levels compared myofibroblasts.		[76]
iPSC-derived systems	*TGFBR3 TGFB2*	Heterozygous knockout and missense variant c.826 G > A; p.G276R	KO (N/A) and G276R (M)	*TGFB2*^+/-^: increased expression of VSMC markers in co-culture condition with *TGFB2*^+/+^ CPC-VSMCs, improved CPC-VSMC differentiation and marker expression upon *TGFB2* supplementation. Similar rescue effects in 3D tissue rings (elongated morphology, improved marker expression). Double mutant *TGFB2*^+/-^ *TGFBR3*^-/-^: impaired differentiation and VSMC marker expression, even with *TGFB2* supplementation. Similar defects in 3D tissue rings (weak signal for VSMC markers, abnormal round VSMC morphology), unchanged cell density. *Patient*^*G276R/+*^ *and TGFB2*^*G276R/+*^: comparable rescue effects of CPC-VSMCs (co-cultures or TGFB2 addition), defective mechanical properties in 3D tissue rings (lower tensile strength).		[77]
	*SMAD3*	Homozygous frameshift variant c.625delA; p.(N218Tfs*23)	M	Engineered iPSC line CPC-VSMC: defective VSMC differentiation, reduced expression of VSMC-specific markers, impaired contractile function and elastin synthesis. Increased collagen deposition, structural stiffness and higher failure force in 3D (tissue rings). Reduced TGF-β canonical pathway activation and responsiveness. CNC-VSMC: unaffected differentiation and VSMC-specific marker expression, significantly reduced elastin synthesis, comparable TGF-β canonical pathway activation and responsiveness to WT cells.		[27]
	*TGFBR1*	Homozygous and heterozygous missense variant c.688 G > A; p.A230T	Patient (F) A230T (N/A)	Patient-derived and engineered iPSC lines *TGFBR1*^*A230T/A230T*^, *TGFBR1*^A230T/+^ and *Patient*^*A230T/+*^: defective differentiation, reduced expression of VSMC-specific markers, impaired contractile function and activation of TGF-β pathway only in CPC-VSMCs, compared to CNC- and WT-VSMCs.		[29]
	*TGFBR1*	Heterozygous missense variant c.688 G > A; p.A230T	Patient (F) A230T (M)	Patient-derived and engineered iPSC lines *TGFBR1*^*A230/+*^ and *Patient*^*A230/+*^: CPC-VSMC-derived bioengineered vascular grafts had impaired mechanical properties (low burst pressure and tensile strength), progressive dilatation after implantation in mice, defective vascular contraction and collagen deposition, reduced thick collagen fibers content, downregulated expression of elastin-associated genes.		[78]

^a^Exons listed in the variants column are included if specified in the original study. These refer to the location of (1) genomic targets for knockouts/knockins, (2) truncating variants for frameshift or nonsense mutations, or (3) deletions/excisions upon loxP cassettes insertion for conditional and tissue-specific alterations. Tissue-specific knockouts are indicated by their respective Cre recombinase driver. For variants reported only at the DNA level (c.) in the original study, the corresponding protein-level changes (p.) were predicted using Franklin software (Genoox). Original annotations and conversions are relative to the following reference sequences: Human, Ensembl transcript ENST00000212345 (GENCODE v43, GRCh38), corresponding to protein ENSP00000212345; Mouse, Ensembl transcript ENSMUST00000112345 (Ensembl v107, GRCm39), corresponding to protein ENSMUSP00000107998; Zebrafish, Ensembl transcript ENSDART00000123456 (Ensembl v107, GRCz11), corresponding to protein ENSDARP00000123456

^b^Unless otherwise indicated in the phenotype column, the gender of experimental animals/cell lines was not reported in the original studies and is marked as N/A. In cases where it was reported but phenotypes were not analyzed separately, no gender-specific differences were observed. Thus, the described phenotypes reflect combined observations from both females and males

ATW, apical terminal web; BL(S), Black Swiss; CNC, cardiac neural crest; CPC, cardiac progenitor cell; DN, dominant negative; ECM, extracellular matrix; EC, endothelial cell; F, female; HAoSMC, human aortic smooth muscle cell; HI, haploinsufficiency; iPSC, induced pluripotent stem cell; M, male; MMP, matrix metalloproteinase; N/A, not available; NC, neural crest; OFT, outflow tract; VSMC, vascular smooth muscle cell; WT, wild-type

##### TGF-β ligand models

Consistent with the fundamental role of TGF-β signaling in cardiovascular development, homozygous knockout (KO) of LDS-associated genes often results in significant embryonic lethality. Roughly two-thirds of *Tgfb2-*null mice die *in utero* or perinatally with severe developmental defects, and the live-born mutants still die due to airway collapse [35]. At embryonic day 18.5 (E18.5), fetuses display multiple congenital heart anomalies (hypoplastic ascending aorta, patent aortic valve leaflets, ventricular septal defects, hypercellular myocardium and hypoplastic right ventricle), as well as craniofacial and skeletal defects (reduced bone size and cranial ossification, morphological mandible abnormalities, cleft palate, limb laxity, sternum and rib malformations). Eye and ear abnormalities were also observed, including hypercellular posterior eye chamber, hyperplastic retinal layers, spiral limbus malformation and incomplete canalization of the cochlea. Cardiac, skeletal, craniofacial and cochlear phenotypes were fully penetrant, although not all mutants exhibited the same cardiac anomalies. The type of defects varied, with ventricular septal defects being the only lesion consistently observed in all animals [35, 36]. The latter findings were observed on mixed genetic backgrounds as well as on C75BL/6 knockout-first and systemic conditional alleles [37].

*Tgfb2*^+/-^ mutants are viable without developmental malformations, but they show signs of LDS4-associated vasculopathy. In the study of Lane et al. [41], male and female C57BL/6 *Tgfb2*^+/-^ mice displayed shorter aortas with reduced aortic curvature and significant wall thickening in the ascending aorta between 4 and 6 months of age. In addition, biomechanical testing further revealed a reduction in biaxial stiffness and stored energy. Interestingly, this wall thickening was not accompanied by any form of dilation or rupture. Conversely, Lindsay et al. [34] demonstrated that *Tgfb2*^+/-^ mice on a mixed background showed a tendency toward aortic root and annulus dilatation starting from 4 months of age, progressing to significant aneurysms by 8 months. These findings suggest that the absence of aortic dilation in the former study may reflect both age- and strain-related progression. Histology of 4-month-old mutants further revealed increased collagen deposition and disorganized elastic fibers within the medial layer [34]. Collectively, the use of homozygous T*gfb2*-null models is limited by significant embryonic lethality, restricting their ability to capture patients’ vascular traits, particularly since most patients harbor heterozygous variants. Heterozygous models align more with the aortopathy of LDS4 patients [1], particularly aortic root dilation, highlighting the predisposed region and affirming that loss of one *TGFB2* allele is sufficient to induce aneurysmal disease. Palatal defects are also well-captured in homozygous mutants, though with strain-dependent penetrance (present in 129 mice, but not in C57BL/6 and Black Swiss strains), suggesting potential interactions between TGF-β ligands and cleft palate susceptibility genes [35, 79].

Similar to *Tgfb2* models, *Tgfb3*^-/-^ mice die perinatally [38, 39]. *Tgfb3*^-/-^ pups of Kaartinen et al. [38], on both C57BL/6 and BL(S) backgrounds, displayed abnormal development of the pulmonary airways and died within 11-20 h after birth. However, the cause of early lethality was not clearly identified, limiting direct comparison with *Tgfb2* mutants in terms of lethality mechanisms. A consistent feature among *Tgbf3*-null mice was palatal clefting, a hallmark of LDS (Tables 1 and 2). These palatal defects, although not accompanied by any other craniofacial manifestations, were more severe and penetrant in C57BL/6 mice compared to 129/Sv and CF-1 strains, suggesting strain-dependent interactions between *Tgfb3* and other cleft palate susceptibility genes.

A more recent study encompassed a detailed cardiovascular assessment of *Tgfb3*^-/-^ mutants on a C57BL/6 background, revealing that around two-thirds of homozygous fetuses developing at least one cardiovascular malformation between E13.5-E18.5 (outflow tract (OFT) septal and alignment defects, abnormal right ventricular myocardium, pulmonary and aortic valve thickening, and abnormal pulmonary and ascending aortic trunk with disorganized VSMC distribution); features comparable to those observed in *Tgfb2*^-/-^ embryos. In contrast, *Tgfb3*^+/-^ fetuses at E14.5 exhibit only mild cardiovascular defects, including hypoplastic vascular walls of the aorta and/or pulmonary trunk, as well as mild thickening of the aortic and/or pulmonary valves [40].

Taken together, heterozygous ligand (*Tgfb2/3*) KO models appear to result in milder cardiovascular anomalies compared to those observed in LDS4/5 patients. By contrast, homozygous KO often leads to more severe congenital heart disease than typically seen in *TGFB2/3* variant-harboring individuals. Nevertheless, these homozygous models may offer valuable insights into the pathogenesis of rare bi-allelic variants, which can produce markedly more severe disease. Rare homozygous *TGFB3* variants have been reported in patients with pronounced craniofacial and musculoskeletal anomalies accompanied by adolescent-onset aortic aneurysms [80, 81]. Moreover, a cleft palate is consistently observed in mutant mice, yet it is only occasionally reported in patients. Possible explanations for these discrepancies include (1) the inherent variability of LDS4/5 expressivity and penetrance in humans, rendering it more challenging to model in mice, (2) the influence of mouse strain background, which modulates phenotypes and (3) the absence of certain features (such as vascular or skeletal features) in some studies, reflecting limited or non-systematic phenotypic assessment rather than true phenotypic divergence.

##### TGF-β receptor models

Homozygous KOs of TGF-β receptors also lead to severe embryonic lethality due to disrupted vascular morphogenesis, particularly the vascularization of the yolk sac [82, 83]. In contrast, heterozygous receptor KO mice are viable and lack overt vascular or craniofacial abnormalities. Renard et al. [43] showed that mice harboring a heterozygous *Tgfbr1* nonsense mutation (p.Y378*) were morphologically and immunohistochemically comparable to WT animals at 6 and 12 months of age. This particular variant triggers nonsense-mediated mRNA decay (NMD), supporting clinical observations that haploinsufficiency alone is insufficient to produce LDS1 manifestations, in contrast to truncating variants that escape NMD [84]. Truncating *TGFBR1* variants leading to NMD are linked to multiple self-healing squamous epithelioma (MSSE) [85, 86].

Since KO models are not always faithful to the human LDS1/2 phenotype, knockin (KI) approaches genocopying patient-specific variants have emerged as an alternative, offering more precise disease representation (Table 2). Gallo-MacFarlane et al. developed and characterized several LDS1/2 mouse models, namely *Tgfbr2*^G357W/+^ and *Tgfbr2*^M318R/+^ mice on a 129S6/Sv background, modeling variants originally identified in severely affected patients [25, 26]. These mutants closely mimicked key LDS traits, including progressive aortic root dilation detectable from 4 weeks of age onwards, leading to pronounced divergence from WT, accompanied by collagen deposition and elastic fiber fragmentation by 24 weeks. Elongation and tortuosity of the aortic and coronary arteries were also observed, along with a predisposition to aortic dissections and early death from hemothorax or hemopericardium. Beyond vascular pathology, skeletal (kyphosis) and craniofacial (craniosynostosis) anomalies were also noted, with craniosynostosis occurring slightly more frequently in *Tgfbr2*^G357W/+^ mutants (7/9) compared to *Tgfbr1*^M318R/+^ mutants (7/11) [25]. These *Tgfbr2*^G357W/+^ craniofacial anomalies were further analyzed quantitatively on the same 129 background, validating the previously reported variability in penetrance and severity, as well as marked left-to-right cranial asymmetry (anterior skull shortening, cranial and palatal suture fusion, cranial doming … etc.), with certain features exhibiting greater severity in female mutants indicative of sexual dimorphism [49].

Similar findings were reported by Yamada et al. [42] in *Tgfbr1* KI mice. Significant embryonic lethality was observed in *Tgfbr1*^*G188V/G188V*^ mutants, with only one surviving to postnatal day 1. At E14.5, embryos had reduced bloodstreams in craniofacial, trunk and spinal cord regions despite the absence of gross patterning defects. *Tgfbr1*^*G188V/+*^ mutants developed a deteriorated aortic wall structure with torn elastic fibers by 24 weeks and showed a tendency of premature death (~90% of mutants surviving until day 180). Furthermore, *Tgfbr1*^*G188V/+*^ mutants showed greater susceptibility to *P. gingivalis-*induced periodontitis, characterized by systemic inflammation and increased alveolar bone resorption, paralleling the increased periodontal vulnerability of LDS patients [87].

Heterozygous KI *Tgfbr2* mouse models present with similar features to *Tgfbr1* KI mice, reflecting the comparable phenotypes observed in humans (Tables 1 and 2). While Gallo-MacFarlane et al. [25] focused on characterizing the vascular, skeletal and craniofacial traits of *Tgfbr2*^*G357W/+*^ mice, the study of Dewan et al. [48] was the first to use MicroCT to assess the morphology of the cortical and trabecular bone of the femur. They identified important abnormalities in cortical bone architecture characterized by a reduced cross-sectional tissue area, bone area and thickness at 6 weeks of age, and persisting until at least 24 weeks. Trabecular bone showed no major morphological changes, except for a modest decrease in mineral apposition rate. Further mechanical testing revealed that 12-week-old *Tgfbr2*^*G357W/+*^ mice had decreased bone strength (lower ultimate moment and bending rigidity) and reduced fracture resistance (lower toughness and post-yield toughness). Snider et al. [88] used 3D morphometry modeling approaches to analyze the postnatal craniofacial morphology of mice with an Osx-Cre-mediated *Tgfbr2* deletion, showing several mineralized tissue anomalies affecting the anterior skull and skull base, palate, mandibular and molar root morphology. Variants in both receptor genes have also been associated with immune-related diseases stemming from disrupted TGF-β signaling. Laky et al. [47], showed that *Tgfbr1*^*M318R/+*^ and *Tgfbr2*^*G357W/+*^ mice spontaneously develop a fully penetrant phenotype closely resembling human eosinophilic esophagitis. The esophagi of these mutants were dilated and immunohistological stainings revealed an increased number of eosinophils, basal cell hyperplasia, abscesses and rete peg (epithelial projections) expansion. Gene expression analysis of *Tgfbr1*^*M318R/+*^ esophageal tissues further highlighted a distressed state characterized by hyperproliferative esophageal epithelial cells with impaired differentiation potential, as well as increased gene expression of cytokines and chemokines. These two KI models were recently shown to capture the extreme enamel defects seen in LDS2 patients, but not in patients with LDS1 [4]. While *Tgfbr1*^*M318/+*^ mice retain normal enamel rod decussation pattern, both female and male *Tgfbr2*^*G357W/+*^ mice exhibit fully penetrant enamel defects characterized by disrupted enamel rod decussation [45]. These defects have been demonstrated to result from impaired coordinated movement of enamel-producing cells (i.e., ameloblasts) during the secretory phase, arising in part from cytoskeletal disorganization and aberrant tight junction distribution within the apical terminal web (ATW) [45, 46].

Inducible tissue-specific KO models are widely employed to study organ-specific disease processes while circumventing the pronounced lethality of germline deficiency. This approach is particularly valuable in the aorta, where conserved regional differences in VSMC embryonic origin have been documented across species, including humans and mice: lateral plate mesoderm (LPM)-derived VSMCs populate the aortic root, whereas cardiac neural crest (CNC)-derived VSMCs contribute to the ascending aorta and aortic arch [89–91]. Within the various LPM-derived subpopulations, cardiac progenitor cells (CPCs) represent a heterogeneous group of multipotent cells that gives rise to the majority of the amniote heart. A subset of these CPCs, the secondary heart field (SHF), contributes to multiple cardiac and vascular lineages, including aortic VSMCs [92, 93].

VSMC-specific deletion of *Tgfbr1* in 9-13 week-old C57BL/6 mice greatly impacts aortic integrity, producing a fully penetrant ascending aortic aneurysm phenotype, with ruptures observed as early as day 10 post-ablation and ~30% associated mortality by day 28 [28]. In contrast, alike *Tgfbr2* deletion led to a mild aortopathy at low prevalence [28]. Similar mild findings, along with vasomotor dysfunction, were reported in *Tgfbr2* mutant mice induced at 11 months of age [53].

It is important to highlight that VSMC-specific *Tgfbr2* loss appears to be age-dependent. Early deletion induced in 4-week old mice leads to severe aortic pathology at 4-6 weeks post-ablation, characterized by consistent ascending aortic dilations, progressing to dissections and hemorrhages. Histologically, medial thickening, elastin fiber fragmentation, matrix accumulation and immune cell infiltration are evident [50, 51].

The VSMC-specific null models also present with abnormalities in neighboring aortic cell types, highlighting the importance of non-cell-autonomous processes. Early (6 weeks) homozygous VSMC-specific deletion of *Tgfbr2* in C57BL/6J mice led to significant VSMC hypercontractility upon stimulation and severe endothelial dysfunction observed as early as 1 week post-deletion [52]. This dysfunction is marked by impaired aortic acetylcholine-induced relaxation and reduced levels of nitric oxide (NO) and endothelial nitric oxide synthase (eNOS), without impacting endothelial permeability or blood pressure. Importantly, this enhanced contractile response is also observed upon NOS inhibition by L-NAME, implicating NO deficiency as a major contributor.

These studies underscore the critical role of TGFBR2 in maintaining vascular homeostasis postnatally. However, it has also key functions during cardiovascular development. These developmental requirements have been demonstrated by selectively disrupting *Tgfbr2* in specific progenitor cells. Conditional homozygous deletion of *Tgfbr2* in NC cells results in fully penetrant cardiovascular abnormalities at E12.5 (persistent truncus arteriosus, absent aortic-pulmonary septum and interrupted aortic arch) and craniofacial defects resembling those observed in TGF-β ligand KO models (i.e., secondary palate clefting). Homozygous *Tgfbr2* deletion in SHF progenitors leads to embryonic lethality at E12.5, with dilation of the OFT and retroperitoneal hemorrhages. Although comparing these models with respect to aneurysm predisposition remains challenging due to the significant lethality, together they demonstrate that both SHF- and NC-derived lineages rely on intact TGFBR2 signaling to preserve vascular integrity during embryonic development.

LDS receptor KO and KI mouse models also recapitulate the gender and interpatient variability in vascular disease frequency and onset. Typically, male patients experience an earlier onset, whereas female patients exhibit a more aggressive disease course and outcome [44]. Homozygous postnatal VSMC-specific deletion of *Tgfbr1* in C57BL/6 mice between 7 and 15 weeks of age revealed fully penetrant aortopathy 28 days post-deletion in males, predominantly affecting the ascending thoracic aorta and leading to premature death, with aortic ruptures accounting for 26% of deaths. Female *Tgfbr1*^*VSMCko*^ mutants did not show any sign of gross aortopathy, with occasional layer tearing only and no early death. This sex-effect was further examined through ovariectomy or orchiectomy. Orchiectomies led to a similar ascending aortic aneurysm phenotype as previously seen in non-castrated mice, with an increased prevalence of proximal and distal descending aortic lesions (dissection, intramural hematoma, and/or fusiform dilation). The aortic rupture and/or dissection prevalence remained comparable between both groups. In contrast, female mice that underwent ovariectomy showed worsened ascending aortopathy, evidenced by intimal/medial layer degeneration, adventitia thickening, elastic fiber breaks and intramural dissections [44].

Endogenous sex hormones thus modulate the aortic aneurysm phenotype of *Tgfbr1*^*VSMCko*^ mutants, with female hormones exerting a protective effect and male hormones leading to an extension of the aneurysmal segment. Furthermore, phenotypic variability is strongly influenced by genetic background. The vascular phenotype of LDS1 mice develops earlier in life on a 129S background compared to C57BL/6 or mixed 129S/C57BL/6J backgrounds [94]. Backcrossing 129S *Tgfbr1*^M318R/+^ mice to a C57BL/6 strain more than two generations led to the development of truncus arteriosus, making them non-viable.

Taken together, homozygous KO *Tgfbr1/2* models highlight the crucial role of TGF-β signaling during early embryonic development, but their use in LDS pathogenesis remains limited due to severe *in utero* or perinatal lethality. On the other hand, heterozygous KI models based on patient-specific variants provide a more faithful representation of LDS features, especially in replicating key vascular phenotypes, ascending aorta regional susceptibility and sex-differences. While vascular and skeletal phenotypes are well-characterized in these models, craniofacial traits are only occasionally examined, leaving the mechanisms driving these defects poorly understood.

##### TGF-β downstream effector models

*Smad2*^-/-^ mice on a mixed C57BL/6 × BL(S) background die early (<E8.5), because of abnormal gastrulation and defective mesoderm induction (Table 2). *Smad2*^+/-^ mutants by contrast, are viable and comparable in growth and size to WT animals [57]. NC-specific C57BL/6 *Smad2*^-/-^ mice survive, but their NC cells aberrantly differentiate into VSMCs within the aortic arch arteries, leading to thinning of the carotid artery wall [58].

Collectively, these findings underscore the essential and cooperative roles of SMAD2/3 signaling in maintaining vascular integrity and proper vascular development. Nevertheless, the limited data available from *Smad2-*deficient mouse models makes it difficult to determine whether they accurately replicate LDS6.

Although *Smad2* and *Smad3* are highly homologous, targeted deletion of *Smad3* in mice does not cause embryonic lethality or any patterning defects. Both *Smad3*^+/-^ and *Smad3*^-/-^ animals are viable and progress through embryogenesis comparably to WT mice [59–61]. However, multiple studies have reported that *Smad3*^-/-^ mice on mixed or 129 backgrounds are smaller in size compared to heterozygous or WT littermates, indicative in some cases of a wasting syndrome [59–61]. Importantly, *Smad3* deficiency is associated with a distinctive immune-driven phenotype characterized by systemic immune dysregulation and inflammation spread throughout the abdomen (inflammatory lesions in the pancreas, stomach, small intestine and colon) [61], or in other cases, formation of colorectal tumors [59].

Beyond these immunopathological features, *Smad3*^-/-^ mutants develop aggressive, early-onset aneurysms of the aortic root and ascending aorta, often leading to sudden death due to aortic dissections, ruptures with cardiac tamponades [24, 61, 62, 64, 65]. A key feature across studies is that the aneurysmal phenotype of *Smad3*^-/-^ mice occurs in the context of inflammation [64, 65]. In the study of Ye et al. [64], aortic root dilation of C57BL/6 *Smad3*^-/-^ mice is evident from 2 months of age and progresses to dilation and/or rupture of the ascending aorta between 4 and 8 months. Remarkably, the tendency to rupture was observed to be independent of the aortic diameter. Early inflammatory changes were present in the aortas of 1-month old mice. Dense infiltration of immune cells (macrophages/monocytes, neutrophils and CD4^+^ T cells) was observed in the aortic roots of *Smad3*^-/-^ mutants, in contrast to a mild infiltration in *Smad3*^+/+^ age-matched littermates. Additional inflammatory lesions in the coronary arteries and immune infiltration of the aortic valve were also reported in mutants. The role of inflammation in the onset of aneurysms was further explored by transplanting *Smad3*^+/+^ bone marrow into *Smad3*^-/-^ recipients, or by depleting CD4^+^ T cells in *Smad3*^-/-^ mutants. While the bone marrow transplant suppressed inflammation and elastic lamina degradation in *Smad3*^-/-^ mutants, the depletion of CD4^+^ T cells resulted in a significant reduction of immune cell infiltration at 8 weeks of age. These findings highlight a pivotal role for adaptive immunity in driving aneurysm formation and progression in *Smad3-*deficient mice.

Sex-specific effects have also been described. While females and males develop similar aortic phenotypes, female *Smad3*^-/-^ mutants exhibit a greater increase in aortic root diameter (21% vs. 14% in males) at 6 weeks of age. However, mortality is more pronounced in males, with 70% dying before 18 weeks of age compared to 50% of female mice [65]. Histological analysis showed that the aortic architecture of these mutants is significantly disrupted, characterized by aortic wall thickening, abnormal internal elastic lamina and elastin degradation without accumulation of ECM proteins including collagen. In line with earlier observations, increased adventitial infiltration (macrophages and CD3^+^ T cells) was evident in 3–4-month-old *Smad3*^-/-^ mice.

In contrast to the severe aortopathy of *Smad3*^-/-^ mice, *Smad3*^*+/–*^ mice do not exhibit a major phenotype at a young age, but have been reported to develop mild aortic root dilation by 8 months of age, suggesting a gene dosage requirement in maintaining aortic wall homeostasis [64].

Similar to constitutive *Smad3*^-/-^ mice, VSMC-specific 129S6/SvEv *Smad3*^-/-^ mice have a severe aortic root phenotype 18 weeks post-deletion, as well as abnormal expression of VSMC focal adhesion proteins and ECM components, such as elastic fibers and contractile proteins, ultimately resulting in the loss of VSMC attachment to the elastic lamellae. No abnormalities have been described in VSMC-specific *Smad3*^+/-^ mice [24, 64].

With respect to the skeletal phenotype, prominent osteoarthritis has been demonstrated in homozygous mutants [62, 63], a feature initially established as a key hallmark of LDS3 [9]. However, recent clinical reports have also identified *SMAD3* patients without overt osteoarthritic manifestations [95, 96]. *Smad3*^-/-^ mice also display forelimb malformations and/or kyphosis [60]. While the inter-vertebral disc of 10-day-old mutants is unaffected, significant degenerative changes occur between 1 and 2 months of age, including spinal malformation and kyphosis, chondrocyte disorganization in the cartilage endplate, reduced type II collagen and proteoglycan content along with increased type X collagen, and calcification [62].

Female and male C57BL/6N *Ipo8*^-/-^ mice, develop mild dilatation of the aortic root between 8 and 12 weeks of age, while severe dilatation of the ascending segment develops in males only (>12 weeks). 30% of males experience aortic ruptures between 32 and 46 weeks of age, compared to none of the females. Histological examination of the male aortas revealed ECM deterioration, with maintained collagen content but disrupted and fragmented elastic fibers.

In summary, among the TGF-β effector models, *Smad3* models are the most extensively characterized, capturing disease features across multiple organ systems (Tables 1 and 2). In particular, aneurysmal disease and chronic inflammation reported in these *Smad3* mutants accurately mirror key clinical manifestations of LDS3 patients, rendering them valuable preclinical tools despite zygosity differences. However, comparison of aneurysm onset and progression remains challenging due to genetic background variability and incomplete vascular characterization in some models (Table 2). Similarly, assessing strain effects in the context of *Smad2* and *Ipo8* effectors is difficult given the limited number of available models.

##### Mouse models synthesis

Although numerous LDS mouse models have been generated, many remain only partially characterized. Despite the autosomal dominant inheritance pattern of LDS, some heterozygous mutant models exhibit relatively mild phenotypes and survive without major complications, displaying only a subset of the systemic features observed in humans. Bi-allelic conditional deficiency results in more pronounced phenotypes in mice, while germline KO often elicits severe embryonic or perinatal lethality, highlighting the crucial role of TGF-β signaling in the early stages of development. Despite these challenges, various mouse models recapitulate different key aspects of LDS pathology, though no single model encompasses the full phenotypic spectrum (Supplementary Table 1).

The most faithful models remain KI mice harboring patient-specific mutations, especially for studying the development and progression of aneurysmal disease and skeletal traits. Collectively, receptor KI models and *Smad3* KO models replicate LDS features more consistently than models targeting other TGF-β downstream effectors or ligands (Table 2). While male mice generally display more severe aortic pathology, emerging evidence indicates that progression may be faster in females. This accelerated course, however, is rarely addressed or investigated, presenting an important opportunity for future research to address sex-specific disease mechanisms and outcomes.

Lastly, it is crucial to note the lack of standardization in defining thoracic aortic dilation and aneurysm in LDS mouse models. Various criteria have been employed for what constitutes an aortic aneurysm (e.g., percentage increase over baseline, absolute diameter threshold or increase relative to control animals), leading to considerable heterogeneity across literature. This variability is further amplified by the use of diverse imaging techniques (e.g., ultrasound, in situ or ex vivo) of distinct anatomical reference points (e.g., aortic root, ascending aorta, aortic arch). This complicates the comparison of findings between laboratories and hinders meta-analyses, limiting translational relevance of preclinical discoveries. Establishing consensus-driven definitions, measurement protocols and reporting standards is therefore essential to enhance the reliability, reproducibility and interpretability of LDS vascular phenotypes in mouse models. Recent guidelines by Daugherty et al. provide recommendations for the design and execution of experimental mouse studies dedicated to aortopathies [97]. Such guidelines are crucial for minimizing methodological variability and ensuring that observed differences between studies reflect true biological effects rather than inconsistencies in experimental design and/or technical procedures.

#### Zebrafish models

Most LDS zebrafish studies focus on cardiovascular characterization rather than craniofacial or skeletal abnormalities. Although the zebrafish heart has two chambers, the developing myocardium forms two sequential outflow segments: the conus arteriosus (CA) and the bulbus arteriosus (BA), with the latter resembling the mammalian aortic root. Most importantly, progenitor contributions to the zebrafish OFT are comparable to those in humans, with SHF progenitors proximally, and CNC-derived cells contributing distally to the BA [98–100].

Boezio et al. [66] used CRISPR/Cas9 genome editing to investigate cardiovascular development in an LDS1 zebrafish KO model by targeting the two *tgfbr1* paralogs; *alk5a* and *alk5b*. Examination of single *alk5*^-/-^ paralog KO larvae revealed no morphological abnormalities or heart defects. In contrast, *alk5a*^-/-^*;alk5b*^-/-^ mutants displayed abnormal cardiovascular morphology, with a dilated OFT. The dilation of the OFT, defined anatomically by a proximal boundary at the bulbo-ventricular canal and a distal boundary at the bifurcation towards the first aortic arch pair, was evident as early as 54 hours-post-fertilization (hpf) and had doubled in size by 78 hpf. Double-mutant embryos also exhibited a reduced EC migration to the ventral region, failing to form the VA. By 96 hpf, a small fraction (~5%) of mutants displayed a ruptured OFT endothelial layer. VSMC disorganization and ECM dysregulation were also noted. Collectively, these abnormalities disrupted cardiac morphology and blood flow, leading to lethality by 7 days-post-fertilization (dpf).

Chida et al. [67] established an LDS2 model by targeting zebrafish *tgfbr2b,* selected for its higher sequence homology to human *TGFBR2*. The antisense morpholino oligonucleotide-mediated knockdown resulted in developmental abnormalities in ~72% of embryos at 1dpf, including delayed head and tail morphogenesis. By 5dpf, mutants exhibited smaller dislocated eyes, smaller bent bodies, rounded heads and shorter caudal fin compared to controls. In addition to these dysmorphic features, morphants developed cardiovascular defects, most notably pericardial effusion and linearization of the cardiac chambers. These phenotypes were not fully penetrant and were observed either in isolation or in combination.

A *smad3* KI zebrafish model was developed to study the vascular phenotype of zebrafish with variants of unknown significance (VUS) in the frame of variant pathogenicity evaluation [68]. In this zebrafish embryo assay, both the positive control (p.T261I), as well as some of the VUS (e.g., p.V244F) displayed enlarged dorsal aortas at 48hpf. While *SMAD3* has two orthologs in zebrafish (*smad3a* and *smad3b*), both of which are expressed in multiple organ systems during development, including the heart [101–103], only *smad3a* mRNA was injected for this assay. This may have influenced the observed phenotypic outcomes, as targeting both orthologs could have provided a more accurate modeling of *smad3* alterations in zebrafish.

Ziegler et al. [69] investigated both cardiovascular and skeletal zebrafish phenotypes following disruption of *ipo8*, which encodes the sole ortholog of human *IPO8* in the zebrafish genome. Homozygous embryos derived from the incross of heterozygous parents did not show any obvious phenotype, suggesting a compensatory role for stored maternal factors during early development. In contrast, 86% of homozygous mutants born from homozygous parents showed a characteristic elongated, pear-like shape caused by impaired tail bud extension around the yolk and associated with increased embryonic lethality at variable penetrance. These tail elongation defects ranged from a partially absent ventral tail fin to a severely distorted body axis. Cardiovascular defects were prominent, with over 70% of mutants displaying abnormal arterio-venous malformations. The central arteries of these embryos appeared irregular and poorly lumenized, in some cases leading to an absent or aberrant blood flow. In addition, 81% of mutant embryos developed heart edemas by 3dpf.

##### Zebrafish models synthesis

The zebrafish models described above have proven valuable in replicating LDS phenotypes, particularly key vascular abnormalities such as altered aortic size and architecture, in addition to few skeletal traits (Table 2). However, models for many LDS-associated genes are lacking and characterization of available models has focused predominantly on the vasculature, limiting in-depth insight in other affected tissues such as the skeleton and craniofacial structures. An additional challenge arises from the presence of multiple zebrafish orthologs for many human genes (*e.g., smad3a* and *smad3b* for *SMAD3*), such that the alteration of only one ortholog can mask or attenuate phenotypes that depend on complete loss of gene function.

The lack of sex-specific analyses further challenges phenotypic interpretation, especially considering the pronounced human and murine differences highlighted earlier in this review. Although such sex-dependent variation has not yet been reported in LDS zebrafish models, differences have been demonstrated for other pathologies [104–106]. Moreover, vascular comparisons between zebrafish and humans remain somewhat challenging, as many human OFT segments lack direct anatomical counterparts in zebrafish. Nonetheless, functional equivalents have been established. For example, the zebrafish BA and DA correspond to the aortic root/ascending aorta and descending aorta, respectively. The designation of the BA as an analog orted by its cellular composition, its position distal to the ventricle and its function in dampening and directing blood flow to downstream vessels [107–109].

### In vitro models

LDS in vitro models provide valuable tools for dissecting cellular and molecular mechanisms under tightly controlled and accessible conditions. Many models have served simple-to-assemble platforms for drug testing, enabling relatively fast, cost-effective and ethically preferable studies compared to animal studies [29]. Recent advances in cell culture methods and stem cell research have allowed the development of LDS in vitro systems that more closely replicate human and even patient-specific physiological conditions, such as tissue-engineered systems and induced pluripotent stem cell-based platforms, enhancing translational relevance [29, 77, 78, 110]. However, such models are often technically demanding and resource-intensive to establish and maintain.

#### Heterologous and primary patient-derived models

Heterologous cell systems have frequently been used to assess LDS-associated TGF-β signaling alterations, employing reporter gene assays. Cardoso et al. [70] employed a SMAD-responsive luciferase reporter in HEK-239T cells to assess the molecular mechanisms of different LDS *TGFBR1* missense mutations (Table 2). Each variant was co-transfected with a constitutively active *TGFBR1* variant p.T204D (TD), providing maximal activation. Co-expression of these constructs in HEK-293T cells revealed a significant reduction in reporter gene activity for most variants as well as impaired SMAD2 nuclear localization and thus, activation compared to the maximal TD signal. Individual LDS constructs transfected with WT *TGFBR1* displayed residual reporter activity above 50%, suggesting that these LDS-causing variants mediate partial, rather than complete LoF. Similar functional effects were observed for *TGFBR2* missense variants [72, 73].

Ishii et al. [75] also used a HEK-239T-luciferase assay to evaluate the pathogenicity of a *de novo* heterozygous *SMAD3* missense variant p.(C421Y). This variant was identified in a 25-year old male patient presenting with TAA, and is located upstream of the C-terminal Ser423-X-Ser425 phosphorylation motif. While exogenous WT SMAD3 enhanced basal Smad-binding element (SBE) luciferase activity, C421Y-transfected cells failed to exhibit a similar response. Furthermore, mutant cells displayed significantly lower phosphorylation levels of SMAD3 compared to WT cells, implying a LoF effect. Of note, elevated TGF-β signaling has been reported in aortic tissue from LDS patients, as well as in *Tgfbr1*^*M318R/+*^ and *Tgfbr2*^*G188V/+*^ mice [1, 25], despite the LoF characteristics of these variants at the cellular level in vitro.

Beyond HEK293-T cells, VSMCs have been studied. Lino Cardenas et al. [74] sought to identify shared molecular signatures between LDS and other genetic aortopathies using human aortic smooth muscle cells (HAoSMCs) with knockdown of *TGFB2, SMAD3, ACTA2* and *MYH11*. Among the top shared dysregulated candidates were *HDAC9, SMAD4, PTGFR* and *PRKCE*. Network and co-immunoprecipitation analysis further demonstrated that HDAC9 interacts with BRG1 (Brahma-related gene 1) to form an HDAC9–BRG1 complex that associates with nuclear RNAs, most notably MALAT1. This complex binds the proximal promoter regions of key contractile genes (*CNN1, SMTN1, TAGLN*) in a MALAT1-dependent manner, contributing to pathological VSMC phenotypic switching [74, 111].

De Wagenaar et al. [76] explored vascular genotype-phenotype correlations associated with *SMAD3* variants using myofibroblasts transdifferentiated from fibroblasts from patients and controls. The impact of haploinsufficient and dominant negative (DN) *SMAD3* mutations on differentiation capacity, contractile marker expression, ECM formation and TGF-β canonical pathway activation was assessed. Differentiation potential varied by mutation type, with HI *SMAD3* myofibroblasts exhibiting an increased differentiation response in the presence of TGFB1 stimulation compared to DN variants. ECM analysis revealed that fibrillin-1 deposition was disorganized in DN mutants and significantly reduced after a 7-day culture period compared to HI lines. Importantly, initial activation of TGF-β signaling pathway in patient fibroblasts, assessed by SMAD2/3 phosphorylation, was not altered in either condition upon stimulation. While this model effectively distinguished HI and DN effects, it was unable to reclassify a VUS (p.I396T), showing intermediate performance between control and DN lines. Findings were validated to some extent but not completely in patient-derived VSMCs, demonstrating that myofibroblasts do not fully replicate the VSMC phenotype.

The models described above have proven valuable in capturing altered TGF-β signaling signatures of LDS, both in patient-derived and heterologous cells (Table 2). Luciferase reporter assays in HEK-293T cells remain the most widely used technique to demonstrate LoF, but their non-vascular origin limits physiological relevance for more in-depth connective tissue disease research. Similarly, while VSMCs provide a more suitable model, they can only capture the vascular phenotype. Tissue specificity also constrains the use of patient-derived cells, as exemplified by differences between dermal fibroblasts-derived myofibroblasts and primary aortic VSMCs [76].

#### iPSC-derived models

Human induced pluripotent stem cell (iPSC) models represent a versatile and practically limitless resource for studying genetic disorders. In the case of LDS, where significant between-patient variability has been described, such models provide a unique opportunity to explore molecular and cellular mechanisms as well as to perform drug testing in a patient-specific context. Numerous iPSC lines from LDS patients haven been created, validated and published [112–117]. While iPSCs can be differentiated into virtually any cell type, all reported LDS iPSC models have focused on the vascular phenotype, leaving musculoskeletal and craniofacial phenotypes understudied.

Studies of the vascular phenotype have predominantly focused on VSMC monocultures, making them the most extensively characterized aortic cell population [27, 29, 77, 78], despite the well-established contributions of ECs, adventitial fibroblasts and immune cells to TAA pathogenesis [44, 51, 60, 64, 65]. Tang et al. [77] examined the impact of *TGFB2* haploinsufficiency on the differentiation and mechanical properties of VSMCs of CPC origin. A significant decrease in VSMC marker expression was observed in *TGFB2*^+/-^ CPC-VSMCs, indicating impaired differentiation potential. This was also obvious in 3D constructs, termed engineered tissue rings, which were generated by culturing CPC-VSMCs around an agarose mold for 7 days until tissue formation. Here, mutant rings showed an abnormal round morphology and similar reduction in VSMC marker expression. Notably, both 2D and 3D cellular anomalies were rescued by either co-culture with WT CPC-VSMCs or exogenous TGFB2 supplementation. The latter abnormalities and rescue effect were shown to extend to CPC-VSMC tissue rings generated from patient (p.G276R) iPSCs, which exhibited reduced and normal tensile strength in the absence and presence of exogenous TGFB2, respectively.

Lineage-specific investigations demonstrated that a homozygous *TGFBR1* missense mutation (p.A230T) primarily affects CPC-VSMCs, leading to reduced expression of the contractile genes at the mRNA and protein level, and impaired canonical (pSMAD3) and noncanonical (pAKT) TGF-β pathway activation [29]. The latter defects, sparing CNC-VSMCs, highly correlate with the aortic root aneurysmal phenotype seen in *TGFBR1*^*A230T/+*^ patients. More recently, *TGFBR1*^*A230T/+*^ CPC-VSMCs were used to create a 3D bioengineered vascular graft (BVG) by culturing cells on a polyglycolide scaffold for 8 weeks [78]. Mechanical testing of these BVGs revealed that *TGFBR1*^*A230T/+*^ structures exhibit lower pressure burst, ultimate tensile stress and suture retention strength compared to WT counterparts. Further in vivo evaluation included the implantation of these BVG pairs (mutant/control) into the left and right common carotid arteries of 3–4-month-old immunodeficient mice. This revealed the progressive expansion of mutant grafts, with a 30% increase in inner diameter 8 weeks post-implantation. With *TGFBR1*^+/+^ BVGs also displaying slight dilation, indicative of active vascular remodeling, mutant BVGs demonstrated an impaired ability to withstand blood flow-induced hemodynamic stress, leading to significant dilation. Moreover, examination of ECM fibers of 8-week explanted mutant BVGs revealed that while collagen content did not differ, thick-to-thin collagen fiber ratio was significantly reduced. Additionally, expression of collagen-related genes, and key elastic fiber assembly genes such as *ELN*, *FBN1*, *FBLN5* and *EMILIN1* were downregulated in mutant BVGs compared to WT BVGs. However, it is important to note that mature organized elastic fibers were not observed in any condition, suggesting suboptimal or immature iPSC-derived constructs. These findings demonstrate the ability of BVGs to recapitulate key LDS1 features, including impaired mechanical integrity and structural ECM alterations, and highlight how the reduction in thick collagen fibers (collagen type I) could contribute to weaker mechanical performances and pathological dilation.

The lineage-dependent differences described above were also demonstrated in the context of a *SMAD3* homozygous frameshift p.(N218Tfs*23), with CPC-VSMCs displaying an abnormal immature morphology along with reduced mRNA and protein expression of key VSMC markers (ACTA2, CNN, TAGLN and MYH11) compared to CNC-VSMCs, which remained largely unaffected [27]. This SMAD3 deficiency also led to a decrease in elastin synthesis in CNC- and, particularly, CPC-derived VSMCs. For TGF-β signaling, impaired activation of the canonical pathway (pSMAD2) upon TGFB1 stimulation and marked downregulation of downstream targets such as PAI-a and CTGF was mainly observed in CPC-VSMCs, while CNC-VSMCs displayed an active canonical response with elevated PAI-1 expression, as well as increased pSMAD2 levels upon stimulation, reflecting intact responsiveness to TGF-β signaling. In 3D tissue rings, increased collagen deposition was noted compared to WT rings, correlating with higher failure force and structural stiffness upon tensile strength testing.

#### iPSC-derived models synthesis

IPSC research relies on robust differentiation protocols and measures to promote cell maturation. VSMC differentiation protocols typically mimic in vivo developmental signaling events (through stimulation of e.g., TGF-β, FGF, Wnt and BMP pathways), but achieve variable induction efficiency due to relatively short timelines. Subsequent maturation is promoted through biochemical stimulation through serum exposure [78, 110], or mechanical cues such as axial stress or cycling stretching [118], with the latter also serving as a functional readout [77, 78, 110].

Current LDS iPSC models are either CRISPR-engineered in healthy iPSCs or derived from patient somatic cells. Characterization of their differentiated counterparts, via a combination of cellular, molecular and functional assays, has revealed valuable insights in terms of defects in differentiation potential, contractile capacity and gene expression, mechanical properties and canonical signaling. Most importantly, key lineage-related differences have been demonstrated for *TGFBR1* and *SMAD3* variants when comparing CNC- versus CPC-derived VSMCs. However, generation of these progenitors requires precise, dose- and time-dependent exposure to morphogens such as TGF-β, raising uncertainty about how the presence of exogenous TGFB1 in differentiation media influences VSMC function in LDS.

Zygotic discrepancies can also be noted in some models. For instance, LDS-causing *SMAD3* variants are typically found in heterozygous state, yet their in vitro characterization relied on homozygous CRISPR-edited iPSCs, eliciting stronger phenotypes that may misrepresent patient biology [110]. Similarly, sex, an established modulator of vascular phenotype, is often mismatched between mutant and control lines, and iPSC donor sex is sometimes unreported (Table 2). To maximize translatability, iPSC models should be faithful to the patient’s zygosity and be directly compared to isogenic controls, minimizing both sex- and genetic background-related variability.

Culture condition is another critical factor that influences model fidelity. 3D systems, such as tissue constructs, organoids and organ-on-a-chip models, mimic more closely the in vivo microenvironments, especially when combined with (patho)physiological mechanical stimuli. Although current LDS 3D iPSC models remain largely VSMC-based (e.g., tissue rings and engineered vascular grafts), they have accurately reproduced critical cellular pathomechanisms, including ECM remodeling and stress-induced mechanical anomalies. These models therefore represent an important step toward more physiologically relevant modeling of aortic defects. Their translational value, however, could be further enhanced by combining multiple VSMC lineages (CPCs and CNCs), or by incorporating additional relevant vascular cell types to better capture the complex intercellular crosstalk underlying TAA development and progression.

### Comparative analysis of LDS models

The models described in this review offer complementary insights into the pathology of LDS, with differences in phenotype fidelity, experimental flexibility, and translational relevance shaping their respective utility. A comparative summary of these models’ strengths, limitations, and extent of phenotypic recapitulation is provided in Table 3.

**Table 3:** Cross-model comparison of LDS preclinical systems

**Model system**^**a**^	Pathology recapitulation	Translational relevance	Key strengths	Key limitations
Mouse	Almost all organ-systems (vascular, craniofacial, skeletal, immune), as well as signaling alterations	Mammalian physiology, conserved cardiovascular development	Whole-organism context, longitudinal studies	Species differences, prolonged generation time (breeding), low throughput, limited translation upon drug testing, strain-dependent phenotype
Zebrafish	Vascular phenotype, some skeletal features	Conserved cardiovascular development	High-throughput, live imaging, developmental insights, short life-span, longitudinal studies	Species/structural differences, prolonged generation time (breeding), limited insights on late-stage (transparency constraints), limited adult physiology fidelity
iPSC-derived cells^b^	Vascular cell-level phenotypes, ECM and signaling alterations	Human genetic background and physiology	Human physiology, mechanistic insights, personalized models	Moderate-to-low throughput, maturation issues, lack of tissue architecture in monocultures, lack of physiological cues

^**a**^The model system column only includes the major LDS preclinical models, excluding vascular and non-vascular primary models (i.e., heterologous cell systems) that are mainly used to investigate aberrant TGF-β signaling

^**b**^iPSC-derived cells, to date, consist exclusively of vascular smooth muscle cells and are therefore limited to the study of vascular defects

## Pathophysiological insights from preclinical LDS research

LDS is one of several alike heritable connective tissue disorders. Among these, Marfan syndrome (MFS), caused by mutations in the *FBN1* gene that disrupt fibrillin-1 and compromise ECM stability, shares notable clinical cardiovascular and skeletal similarities with LDS. However, since MFS was identified first, it has been more extensively characterized, forming an important foundation for understanding the pathomolecular mechanisms underlying LDS.

Dysregulated TGF-β signaling is central to both syndromes. Exaggerated TGF-β activity, first observed in MFS mouse models through increased SMAD2/3 phosphorylation and nuclear translocation, was later found in vascular surgical samples from LDS patients [119]. In MFS, *FBN1* mutations impair TGF-β sequestration, increasing active TGF-β in tissues, consistent with pathway activation. In contrast, LDS results from L0F mutations in TGF-β ligands, receptors, or downstream effectors, theoretically expected to reduce signaling, making the observed increase counterintuitive.

Although the mechanisms driving this paradoxical upregulation in TGF-β signaling in LDS remain unclear, several potential mechanistic hypotheses have been proposed and are being further investigated. A first hypothesis proposes excessive TGF-β ligand production in response to weakened SMAD-dependent TGF-β signaling feedback inhibition. [120]. This ligand excess may, in turn, drive increased signaling through both residual canonical pathways and non‑canonical pathways, including ERK1/2, MAPK, PI3K/AKT, and Rho/ROCK signaling cascades. Secondly, defective TGF-β receptor internalization or recycling may prolong surface retention and enhance ligand-receptor interactions [121]. Another hypothesis is that crosstalk between canonical and non-canonical branches may create feedback loops that reinforce signaling activity, for instance, MAPK and PI3K/AKT cascades can phosphorylate SMADs at inhibitory or linker sites, fine-tuning their transcriptional output [121, 122].

In addition to altered autoregulation and receptor trafficking, the paradox may also stem from interactions between VSMCs of distinct embryonic origins and their differential responses to TGF-β [26, 121]. Especially the latter hypothesis has been further investigated in recent years. Further examination in the aortas of *Tgfbr1*^*M318/+*^ of the signaling ability of different VSMC types intersecting at the aortic root, i.e., SHF-, and CNC-derived VSMCs, demonstrated that this mutation causes defective Smad2/3 activation, upregulation of AT1R (angiotensin II type 1 receptor) and over-sensitization to AngII only in SHF-derived VSMCs [26]. These SHF-derived cells can influence adjacent CNC-derived VSMCs through paracrine signaling, driving pathological changes beyond their own lineage. This highlights how lineage-specific signaling abnormalities, together with inter-lineage crosstalk, contribute to the regional predisposition to LDS aortopathy [123]. AT1R mediates AngII-driven effects on vasoconstriction, fluid balance, VSMC phenotype, matrix deposition, and inflammation. In line with these findings, a recent study in *Tgbr1*^*M318R/+*^ mice revealed that SHF-specific deletion of *At1r* can rescue to a great extent the previously observed phenotype but remains less beneficial compared to the global deletion of *At1r,* demonstrating that AT1a signaling in non-SHF-derived cells and systemic effects such as blood pressure reduction may also contribute to aortic protection [23]. These lineage-related events are partly captured by iPSC-derived VSMC models. A homozygous *SMAD3* frameshift was shown to increase TGF-β signaling in CNC-derived VSMCs, suggesting a potential mechanism contributing to proximal aortic aneurysm formation. In contrast, the same homozygous LoF variant impaired canonical TGF-β signaling during CPC-VSMC differentiation, underscoring the SMAD3 dependency of VSMCs populating the aortic root [27]. Such divergent responses between lineages may not act in isolation; crosstalk or paracrine signaling between CPC- and CNC-derived VSMCs could amplify or modify these effects in vivo.

Uncertainty about the TGF-β paradox led to an ongoing debate regarding whether TGF-β signaling is primarily protective or deleterious in LDS pathogenesis. On one hand, excessive TGF-β activity associates with aortic aneurysm formation and vascular degeneration, and its attenuation benefits several LDS models [25]. On the other hand, complete loss of TGF-β signaling causes severe developmental and immunological abnormalities, indicating its essential role in maintaining vascular and tissue homeostasis [124]. These opposing effects likely reflect a context- and time-dependent function of TGF-β, varying with disease stage, cell type and the microenvironment.

The advent of next-generation sequencing has enabled hypothesis-free LDS investigation beyond TGF-β dysregulation, particularly through bulk and single-cell RNA sequencing (scRNA-seq). ScRNA-seq of aortic tissue from LDS patients and mouse models (Table 4). revealed substantial cellular heterogeneity and lineage-specific effects, especially among VSMCs and fibroblasts. Apart from variable expression of canonical TGF-β target genes (e.g., *Serpine1, Ctgf, Col1a1, Col1a2*) in *TGFBR1*-mutant VSMCs, CPC-derived VSMCs showed reduced contractile markers expression (*ACTA2, CNN1, SM22α, MYOCD*), while CNC-VSMCs were less affected [29, 126]. Notably, adventitial fibroblasts exhibited increased inflammatory (*Ccl2, Il6, Ccl7, Cxcl2*) and fibrotic (*Col1a1, Col3a1*) gene expression, correlating with macrophage infiltration and tissue remodeling [126]. Unique SHF-derived fibroblast subclusters also exhibit decreased ECM gene expression (*Eln, Col3a1*), potentially compromising aortic wall integrity [127]. Complementing these findings, bulk RNA-seq has identified LDS-specific gene expression profiles marked by upregulation of inflammatory and fibrotic pathways [47].

**Table 4:** Overview of RNA-sequencing datasets in Loeys-Dietz syndrome

Type	Model	Gene(s)	Age	Sex	Tissue(s)	Accession	Ref
scRNA-seq	Human	*TGFBR1/2, TGFB2 and SMAD3*	26–51 years	M & F	Ao, AAo	N/A	[125]
	Human	*TGFBR1/2*	Various	M & F	Ao, AAo	GSE235678	[126]
	Mouse (129S6/SvEv)	*Tgfbr2*^*G357W/+*^	24 weeks	M & F	Ao, AAo	GSE235638	[126]
	Mouse (129S6/SvEv)	*Tgfbr2*^*G357W/+*^	8 weeks	M & F	Ao, AAo	GSE288084	[126]
	Mouse (129/SvEv)	*Tgfbr1*^*M318R/+*^	16 weeks	F	Ao, AAo	GSE267204	[123]
	Mouse (129S6/SvEv)	*Smad3*^*VSMCKO*^	10 and 18 weeks	M	Ao, AAo	GSE189025	[24]
	iPSC-VSMC	*TGFBR1*^*A230T*^	N/A	N/A	iPSC-derived CPC- and CNC-VSMC	GSE175647	[29]
Bulk RNA-seq	Mouse (129/SvEv)	*Tgfbr1*^*M318R/+*^	16 weeks	M & F	Esophagus	GSE193756	[47]
	Mouse (129S6/SvEv)	*Smad3*^*VSMCKO*^	8 weeks	M & F	SHF- and CNC-cells Ao, AAo	GSE189544	[24]

Ao, aortic root; AAo, ascending aorta; CNC, cardiac neural crest; CPC, cardiac progenitor cell; F, female; iPSC, induced pluripotent stem cell; KO, knockout; M, male; N/A, not available; SHF, secondary heart field; VSMC, vascular smooth muscle cell

**Fig. 3:**
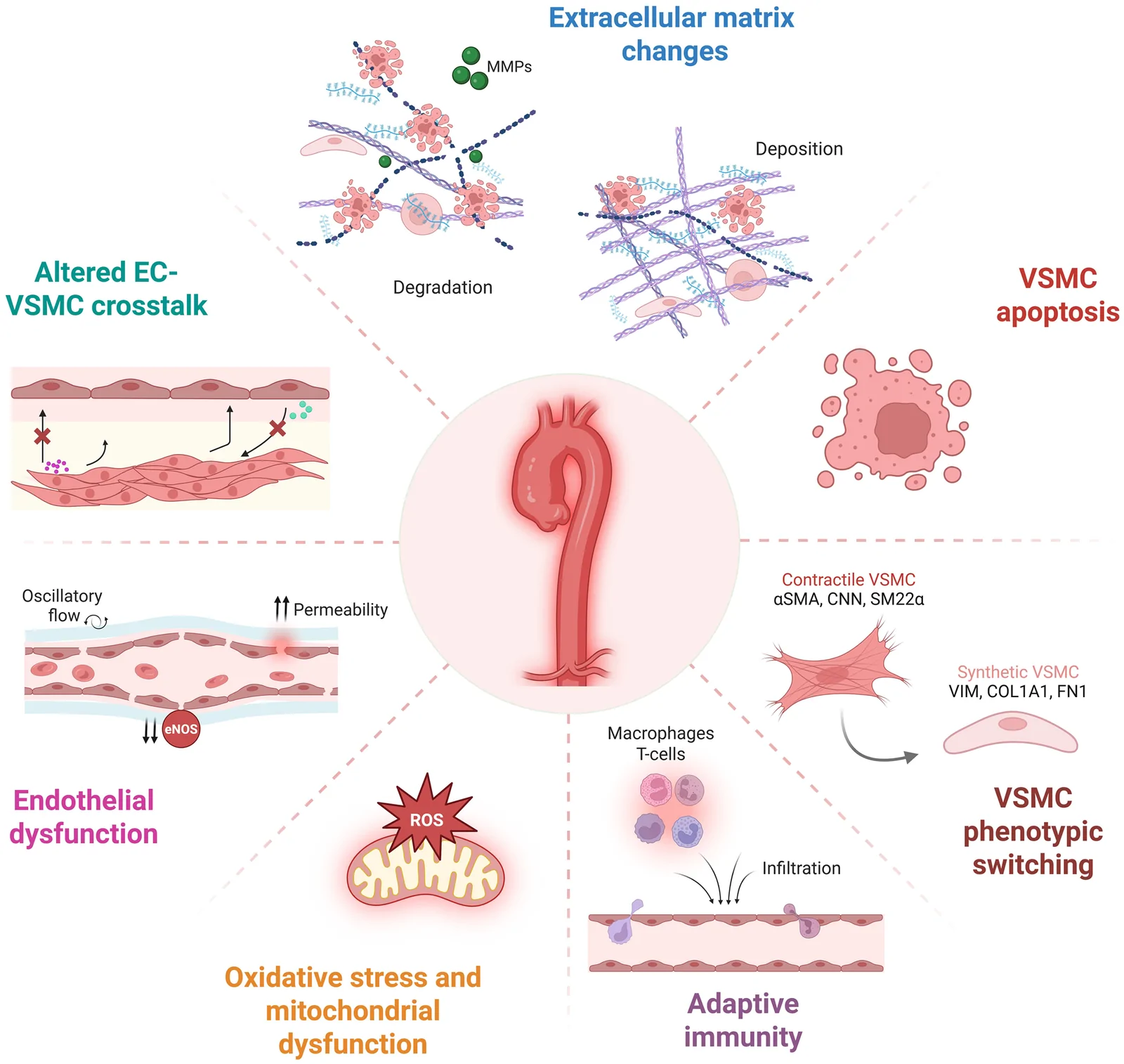
Overview of the cellular and molecular mechanisms in Loeys-Dietz syndrome. Schematic representation highlighting the most extensively studied pathomechanisms. EC, endothelial cell; eNOS, endothelial nitric oxide synthase; MMP, matrix metalloproteinase; ROS, reactive oxygen species; VSMC, vascular smooth muscle cell. Created in BioRender https://BioRender.com/asb4plw

Despite these molecular insights (Fig. 3), LDS research remains heavily aorta-focused, with minimal data on other organ systems or even on non-aortic cardiovascular manifestations, such as the more frequent involvement of mid-sized arteries. The recent identification of adventitial fibroblasts as key contributors to the LDS aortic phenotype underscores the potential of extending molecular investigations beyond VSMCs and even the aorta to better understand LDS.

## Therapeutic insights from preclinical LDS research

The management of LDS has also been significantly influenced by lessons learnt from MFS. The first preclinical study of losartan in an MFS mouse model was by Habashi et al. in 2006 [128]. Since then, several clinical trials for different therapeutic agents for MFS have been completed [129–132], shaping international recommendations for LDS patient management, often without preclinical confirmation in LDS-specific models [20, 22].

Among the available preclinical systems, genetically defined LDS mouse models, particularly those targeting *Tgfbr2* and *Tgfbr1*, have been especially informative and represent the most predictive platform for evaluating pathway‑targeted therapies. Despite that, to date, only a few compounds have had their effects confirmed in LDS, mostly by replicating results initially observed in MFS models. Gallo-MacFarlane et al. showed a significant advantage of an angiotensin-II-receptor blocker (losartan) compared to β-blocker (propranolol) in *Tgfbr2*^*G357W/+*^ mouse model, consistent with previous findings in MFS mice [2, 128]. Unlike losartan, β-blockers failed to preserve aortic wall architecture despite slowing the rate of aortic root dilation. The beneficial effects of losartan in limiting pathologic aortic growth and medial degeneration are likely due its antihypertensive properties and its well-established capacity to modulate excessive TGF-β signaling, which was a key reason for its selection as a candidate drug in LDS mice [25]. In *Tgfbr1*^*iko*^ mice, losartan was demonstrated to lead to blood pressure reduction, inhibition of aortic dilation and normalization of aortic structure [28]. In contrast, hydralazine, an inhibitor of PKCβ-mediated ERK phosphorylation, and the β-blocker propranolol both lowered blood pressure, with propranolol appearing to attenuate aneurysmal degeneration, but neither drug reduced aortic dilation, which remained equivalent to placebo controls, despite prior efficacy in MFS mouse models (hydralazine) and patients (propranolol) [133, 134]. The selection of the PKCβ pathway as a therapeutic target was motivated by its role in ERK activation, which indicates at least an indirect mechanistic link.

Rapamycin, an mTOR inhibitor that suppresses smooth muscle cell proliferation, has effectively prevented aortic dilation and dissection in mice with conditional *Tgfbr2* deletion in smooth muscle cells [51]. Activin A, a member of the TGF-β superfamily, can activate SMAD2/3 signaling to promote contractile phenotype maintenance. Zhou et al. tested a combined treatment with activin A and rapamycin, based on their finding that heterozygous *TGFBR1* mutant CPC-derived VSMCs displayed disrupted SMAD3 and AKT signaling, increased proliferation, and impaired mechanical properties [29]. In vitro, the dual therapy restored the mechanical function of mutant smooth muscle 3D constructs, improving tensile strength and stiffness, suggesting additive benefits from targeting both proliferative and SMAD pathway deficits. However, the in vivo efficacy of this combined approach in the context of LDS remains unknown.

Despite growing pathogenesis insights, few preclinical drug tests have been published, and clinical translation remains limited. No clinical trials have been conducted exclusively in LDS patients. Sandor et al. [135] included cohorts, consisting of LDS and MFS patients, in a pilot study of losartan and atenolol effects on aortic biophysics. However, due to a small number of participants, it was not possible to draw definite conclusions. Moreover, no therapeutic studies have targeted non-cardiovascular LDS manifestations, leaving clinical management largely symptomatic.

## Conclusion and future directions

### LDS-tailored preclinical research

Given the clinical and pathophysiological overlap between LDS and MFS, and MFS’s earlier discovery and higher prevalence, many LDS management strategies have been extrapolated from MFS. While both share features like aortic root aneurysm, pectus deformity, and joint laxity, LDS has distinct genetic and clinical traits requiring special attention (e.g., peripheral aneurysms, arterial tortuosity, hernias, and poor wound healing) [136]. Extrapolating MFS findings to LDS has limitations, as it may overlook LDS-specific molecular and clinical nuances. Fortunately, LDS-specific research is growing, essential for uncovering unique pathways and vulnerabilities, paving the way for innovative treatments.

The increasing availability of iPSC-derived LDS cellular models opens new opportunities for applying high-throughput drug screening approaches to identify novel therapeutic targets. If a robust and scalable readout is established, such as contractility, proliferation, or pathway-specific reporter activity, these systems could be used for systematic perturbation studies. For instance, altered SMAD3 and AKT signaling [29], as well as changes in ECM composition and reduced VSMC marker expression [27], could be used for screening. Similarly, zebrafish models of TGF-β pathway mutations offer an efficient and scalable platform for in vivo drug screening, particularly when vascular or cardiac phenotypes are used as readouts. Incorporating these approaches could accelerate the discovery of LDS-specific interventions and better capture patient-relevant biology.

### Gaps, needs and future developments

Addressing the urgent need for targeted LDS therapies requires models that reflect the disorder’s multisystem complexity. While KO or transgenic mouse and zebrafish models provide valuable mechanistic insights, structural and functional differences in cardiovascular, immune and metabolic systems remain significant limitations. In addition, the role of genetic background in influencing phenotypic variability and therapeutic response is increasingly recognized but remains underexplored. Moreover, key variables such as sex and strain are often omitted in preclinical reports, compromising reproducibility and data interpretation. Comparative analysis across models is further complicated by differences in how disease manifestations are assessed, often at varying timepoints (Table 2). The absence of pathology in certain models may reflect premature evaluation rather than true phenotypic variability, underscoring the need for longitudinal studies to validate phenotypes and clarify mechanisms underlying late-stage vascular disease. iPSC-derived systems, though human-based, often lack the cellular diversity and tissue architecture needed to fully recapitulate in vivo interactions. In this context, the development of vascular organoid and engineered 3D tissue models incorporating patient‑derived iPSC‑VSMCs, endothelial cells and fibroblasts offers a more physiologically relevant platform for pathway interrogation and drug screening, better capturing the multicellular and architectural complexity of the aortic wall, and enabling investigation of additional cell types that are increasingly recognized as critical in LDS pathogenesis. Expanding preclinical studies to encompass other affected organ systems and intercellular interactions will be crucial for developing more accurate and translatable therapeutic strategies for LDS. Finally, we expect the increasing accessibility and affordability of single‑cell and spatial transcriptomic technologies to substantially advance the understanding of cell‑type‑specific gene expression programs within the aortic wall, including the roles of distinct VSMC lineages, fibroblasts, and immune cells, thereby facilitating the discovery of novel drug options.

## Electronic supplementary material

Below is the link to the electronic supplementary material.

Supplementary material 1

## Data Availability

No new datasets were generated or analyzed for this review article. All data discussed are available from the cited publications.

## Abbreviations

ATW: Apical terminal web
BA: Bulbous arteriosus
BL(S): Black Swiss
BVG: Bioengineered vascular graft
CA: Conus arteriosus
CNC: Cardiac neural crest
CPC: Cardiac progenitor cell
DN: Dominant negative
Dpf: Days-post-fertilization
EC: Endothelial cell
ECM: Extracellular matrix
eNOS: Endothelial nitric oxide synthase
HI: Haploinsufficiency
Hpf: Hours-post-fertilization
iPSC: induced pluripotent stem cell
KI: Knockin
KO: Knockout
LDS: Loeys-Dietz Syndrome
L-NAME: L-Nitroarginine Methyl Ester
LoF: Loss-of-function
LPM: Lateral plate mesoderm
MFS: Marfan syndrome
MMP: Matrix metalloproteinase
MSSE: Multiple self-healing squamous epithelioma
NMD: Nonsense-mediated mRNA decay
NO: Nitric oxide
OFT: Outflow tract
ROS: Reactive oxygen species
SBE: Smad-binding element
SHF: Secondary heart field
TGF-β: Transforming growth factor beta
VA: Ventral aorta
VSMC: Vascular smooth muscle cell
VUS: Variant of unknown significance
WT: Wild-type
