# Supplementary material for: Preclinical modeling of Loeys-Dietz syndrome: insights into mechanisms and therapy
Source: Orphanet J Rare Dis. 2026 Jun 30;21:330. doi: 10.1186/s13023-026-04431-1 (PMC13602759; doi:10.1186/s13023-026-04431-1)
Supplement: Supplementary file 1 [file 13023_2026_4431_MOESM1_ESM.docx]

| Supplementary Table 1 Comparison of the different features in Loeys-Dietz syndrome mouse models | | | | | | | |
| --- | --- | --- | --- | --- | --- | --- | --- |
| Systemic features | **Genes** | | | | | | |
|  | *Tgfbr1* | *Tgfbr2* | *Smad3* | *Tgfb2* | *Tgfb3* | *Smad2* | *Ipo8* |
| Embryonic lethality | **+** | **+** |  | **+** | **+** | **+** |  |
| Aortic dilatation | **+** | **+** | **+** | **+** | **+** |  | **+** |
| Arterial tortuosity | **+** | **+** | **+** |  |  |  |  |
| Aortic wall architecture abnormalities | **+** | **+** | **+** |  |  | **+** | **+** |
| Aortic rupture and/or dissection | **+** | **+** | **+** |  |  |  | **+** |
| Structural heart defects |  |  |  | **+** | **+** |  |  |
| Lung developmental defects |  |  |  |  | **+** |  |  |
| Pneumothorax/hemothorax | **+** | **+** |  |  |  |  |  |
| Bifid uvula/cleft palate |  |  |  | **+** | **+** |  |  |
| Craniosynostosis | **+** | **+** |  |  |  |  |  |
| dental anomalies | **-** | **+** |  |  |  |  |  |
| Retinal abnormalities |  |  |  | **+** |  |  |  |
| Auricular abnormalities |  |  |  | **+** |  |  |  |
| Spine instability/abnormality | **+** | **+** |  |  |  |  |  |
| Abnormal bone morphology and/or mechanical strength | **-** | **+** |  | **+** |  |  |  |
| Inflammation and/or immune dysregulation | **+** | **+** | **+** |  |  |  |  |

*(+) indicates the presence of the feature; (-) indicates the absence of the feature and empty cells indicate that the feature was not mentioned or investigated in the model.*

*The systemic features listed in this table vary depending on the affected gene, variant type and genetic strain. Interpretations should be made in conjunction with Table 2, which provides detailed phenotypic information.*
